# Intracellular Staphylococcus aureus Perturbs the Host Cell Ca^2+^ Homeostasis To Promote Cell Death

**DOI:** 10.1128/mBio.02250-20

**Published:** 2020-12-15

**Authors:** Kathrin Stelzner, Ann-Cathrin Winkler, Chunguang Liang, Aziza Boyny, Carsten P. Ade, Thomas Dandekar, Martin J. Fraunholz, Thomas Rudel

**Affiliations:** a Chair of Microbiology, University of Würzburg, Würzburg, Germany; b Chair of Bioinformatics, University of Würzburg, Würzburg, Germany; c Chair of Biochemistry and Molecular Biology, University of Würzburg, Würzburg, Germany; d Helmholtz Institute for RNA-based Infection Research (HIRI), Würzburg, Germany; Harvard Medical School

**Keywords:** *Staphylococcus aureus*, calcium signaling pathway, cell death, facultatively intracellular pathogens

## Abstract

The opportunistic human pathogen Staphylococcus aureus causes serious infectious diseases that range from superficial skin and soft tissue infections to necrotizing pneumonia and sepsis. While classically regarded as an extracellular pathogen, S. aureus is able to invade and survive within human cells. Host cell exit is associated with cell death, tissue destruction, and the spread of infection. The exact molecular mechanism employed by S. aureus to escape the host cell is still unclear. In this study, we performed a genome-wide small hairpin RNA (shRNA) screen and identified the calcium signaling pathway as being involved in intracellular infection. S. aureus induced a massive cytosolic Ca^2+^ increase in epithelial host cells after invasion and intracellular replication of the pathogen. This was paralleled by a decrease in endoplasmic reticulum Ca^2+^ concentration. Additionally, calcium ions from the extracellular space contributed to the cytosolic Ca^2+^ increase. As a consequence, we observed that the cytoplasmic Ca^2+^ rise led to an increase in mitochondrial Ca^2+^ concentration, the activation of calpains and caspases, and eventually to cell lysis of S. aureus-infected cells. Our study therefore suggests that intracellular S. aureus disturbs the host cell Ca^2+^ homeostasis and induces cytoplasmic Ca^2+^ overload, which results in both apoptotic and necrotic cell death in parallel or succession.

## Abstract (precis)

Despite being regarded as an extracellular bacterium, the pathogen Staphylococcus aureus can invade and survive within human cells. The intracellular niche is considered a hideout from the host immune system and antibiotic treatment and allows bacterial proliferation.

## INTRODUCTION

Staphylococcus aureus is a Gram-positive pathogen that frequently colonizes part of the human microflora (1). Despite its common mode of asymptomatic colonization in the healthy population, immunocompromised patients often suffer from serious infection outcomes, which range from superficial skin lesions to deep-seated abscesses and life-threatening sepsis and which are often caused by the endogenous carriage strain (2, 3). S. aureus infections thus lead to high morbidity, and mortality and health care-associated costs have been exacerbated by S. aureus isolates with resistance against many antibiotics (4).

S. aureus has been demonstrated to invade many mammalian cell types, such as epithelial and endothelial cells, osteoblasts, fibroblasts, and keratinocytes (5, 6). The pathogen has been shown to survive inside host cells for extended periods of time, not only *in vitro* (7–11) but also *in vivo*, where intracellular S. aureus has been detected in tissue samples and phagocytic cells (12–16). The intracellularity of S. aureus is associated with chronic or relapsing staphylococcal infections, as well as with resistance to the host immune system and antibiotic treatment.

S. aureus adherence and subsequent uptake by nonprofessional phagocytes is facilitated by several bacterial adhesins, which interact with extracellular matrix proteins or specific host receptors (17, 18). Translocation from the bacteria-containing vacuole to the host cell cytoplasm is required for intracellular replication of the pathogen in, for instance, epithelial cells (19). Infection of cultured cells with S. aureus often elicits severe cytotoxicity that is believed to be the cause of pathologies originating from infection-induced tissue destruction (3, 20). Various rounds of S. aureus internalization and escape from host cells may contribute to tissue and membrane barrier destruction, spread of infection, immune evasion, and inflammation (21–23).

Previous work has demonstrated a fundamental role of staphylococcal virulence factors in cellular cytotoxicity, directly damaging the cell and causing apoptotic and/or necrotic types of cell death (24–26). Besides, S. aureus invasion of host cells was shown to be required to activate cell death mechanisms (27–32). Very little is known about the cellular signaling involved in staphylococcal intracellular infection. To date, studies of host cell death in nonprofessional phagocytes infected with S. aureus provide conflicting results. Hallmarks of apoptosis, such as DNA fragmentation, cell contraction, and/or activation of caspases, were reported in S. aureus-infected epithelial cells (27, 29, 33, 34). In bovine mammary epithelial (MAC-T) cells, the extrinsic apoptosis pathway involving inflammatory cytokines and caspases 8 and 3 was activated by intracellular S. aureus (35). In contrast, induction of autophagy, but not activation of caspases, was shown to be essential for S. aureus cytotoxicity (36). Details on how intracellular S. aureus interferes with cell death signaling remain to be elucidated.

Ca^2+^ signaling has been implicated in various steps of bacterial infection, and several bacterial toxins are known to induce Ca^2+^ fluxes in host cells by the formation of pores in the plasma membrane and/or by activation of Ca^2+^ channels (37). Due to its low cytosolic concentration, calcium ions act as second messengers controlling diverse cellular functions, such as cell survival and cell death (38–41). Increases in cytosolic Ca^2+^ levels originate from uptake of extracellular Ca^2+^ by plasma membrane Ca^2+^ channels or by extrusion of calcium ions from internal stores. Store-operated calcium entry (SOCE) represents the predominant Ca^2+^ entry mechanism in nonexcitable cells. Here, Ca^2+^ depletion from the endoplasmic reticulum (ER) activates Ca^2+^ entry across the plasma membrane and thus refills internal Ca^2+^ stores (42). For instance, regulated Ca^2+^ elevations are involved in cell signaling during the intrinsic apoptotic pathway, and local Ca^2+^ messages at the ER-mitochondrion interface participate in early apoptosis. A family of cysteine proteases called calpains also plays a role in Ca^2+^-induced apoptosis (43). The Ca^2+^-sensitive proteases cleave several members of the caspase and BCL2 family to regulate apoptotic cell death. Furthermore, high concentrations of mitochondrial Ca^2+^ activate mitochondrial permeability transition (MPT)-driven necrosis (44, 45). Disruption of ER Ca^2+^ homeostasis may result in ER stress and unfolded protein response (UPR), eventually leading to cell death (46). When stress results in cellular Ca^2+^ overload or loss of Ca^2+^ homeostatic control, Ca^2+^ functions also as the executer of cell death (39).

In this study, we investigated how intracellular S. aureus interferes with host cell Ca^2+^ signaling. A Ca^2+^ reporter cell line and live cell imaging were used to monitor Ca^2+^ fluxes in single cells infected with the pathogen. Our results indicate that the intracellular bacterium perturbs the host cell Ca^2+^ homeostasis to induce cell death.

## RESULTS

### An unbiased shRNA screen identifies calcium-associated genes and pathways in S. aureus invasion and cytotoxicity.

**FIG 1:**
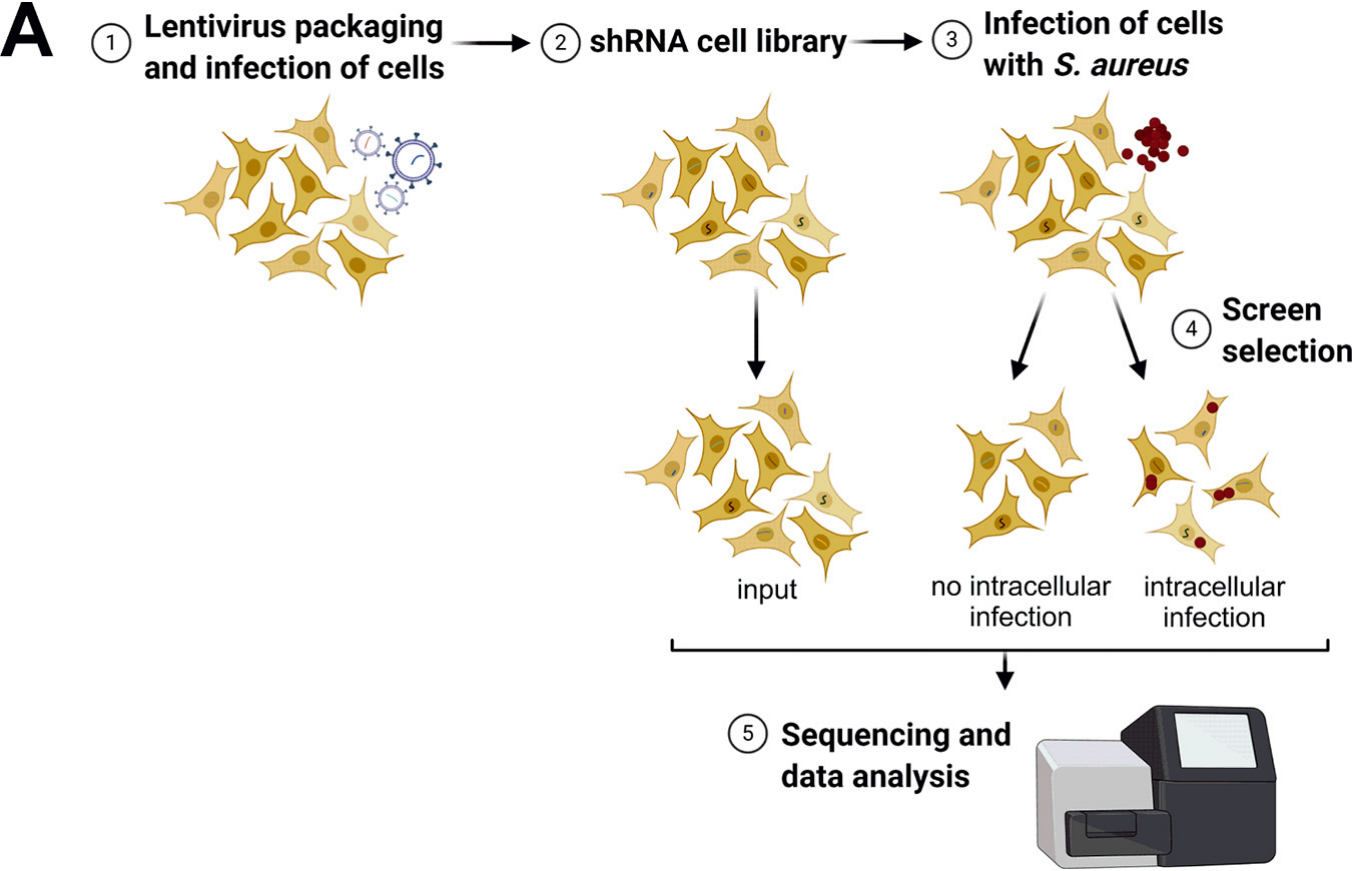

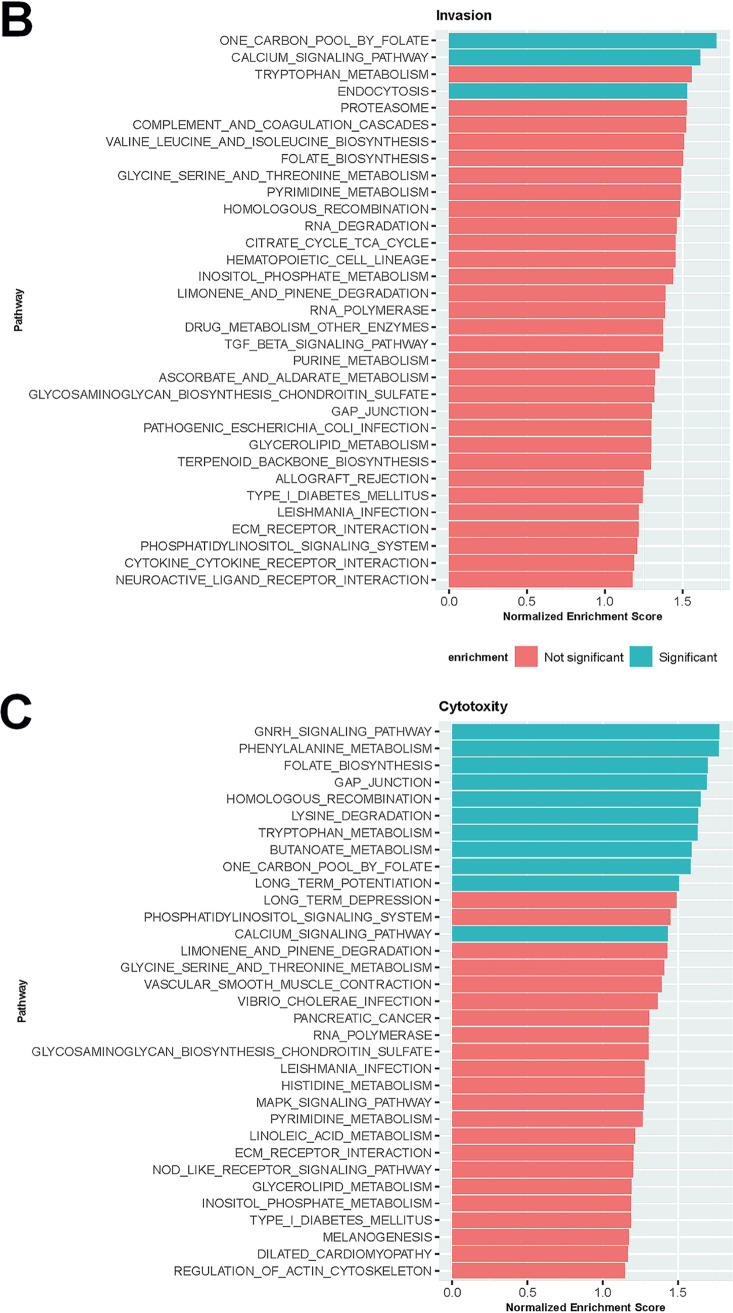
Small hairpin RNA (shRNA) screen to identify host cell genes involved in S. aureus invasion and intracellular cytotoxicity. (A) Layout of shRNA screening approach. HeLa cells were first transduced with the shRNA library. Stable shRNA-HeLa cells were infected with S. aureus 6850 mRFP. At 4.5 hours postinfection (h p.i.), dead cells were removed, and both cells containing no intracellular bacteria and cells with intracellular S. aureus were sorted via fluorescence-activated cell sorting (FACS). Subsequently, genomic DNA was extracted and prepared for Illumina sequencing to determine enrichment or depletion of shRNAs in comparison to input HeLa cells, which were used as a negative control. (b, c) a detailed gene set enrichment analysis (GSEA) of genes identified by the shRNA screen to be implicated in S. aureus invasion (B) or cytotoxicity (C) revealed key pathways. Pathways with a *P* value lower than 0.02 are noted as significant.

We performed a whole-genome RNA interference (RNAi) screen using small hairpin RNAs (shRNAs) to identify host cell genes involved in S. aureus invasion and cytotoxicity (Fig. 1A). HeLa cells were transduced with a lentiviral shRNA library, thereby generating a pool of host cells, in which genes were knocked-down by RNAi. The shRNA library used comprised 52,093 different shRNAs targeting 11,226 different host genes. This pool of HeLa cells (“input”) was infected with S. aureus 6850, a strongly cytotoxic and cell-invasive strain (47), expressing red fluorescent protein (mRFP). After an infection pulse of 1 h, extracellular bacteria were removed by treatment with the staphylolytic protease lysostaphin to ensure that cytotoxicity was not exerted by residual bacteria in the tissue culture medium. Cell populations with and without intracellular S. aureus were subjected to fluorescence-activated cell sorting (FACS) at 4.5 h postinfection (see Fig. S1A in the supplemental material). DNA was prepared from the “input” pool as well as from the cells with and without intracellular S. aureus infection, and individual shRNA frequencies were determined by next-generation sequencing. Quality controls and data normalization were performed, and a principal-component analysis (PCA) demonstrated the validity of the data sets (Fig. S1B and C). Selective enrichment of shRNAs retrieved from cells with and without intracellular S. aureus infection were compared to the input cell pool. We thereby quantified the differential shRNAs (false discovery rate [FDR]-adjusted *P* value < 0.001; log_2_[fold change] [log_2_FC] = 1.5), of which 79 were enriched and 217 were depleted in the sample with no intracellular infection (see Table S1 in the supplemental material), indicative of shRNAs that negatively and positively regulate the bacterial invasion process, respectively. In the sample with cells surviving intracellular S. aureus infection, 233 shRNAs were enriched and 311 were depleted (Table S1), reminiscent of shRNAs that negatively and positively regulate infection-induced cell death. Genes targeted by shRNAs with a log_2_FC of 3 (invasion) or 3.5 (cytotoxicity), respectively, are illustrated in heatmaps (Fig. S1D and E).

10.1128/mBio.02250-20.3 FIG S1 Small hairpin RNA (shRNA) screen. (A) Intact cells were defined by flow cytometric analysis via forward scatter (FSC) and sideward scatter (SSC) (P1). Green fluorescent protein (GFP)-expressing cells, indicating shRNA expression, were gated as P4. Cells harboring intracellular S. aureus 6850 showed increased red fluorescent protein (mRFP) fluorescence. Uninfected and infected HeLa cells were gated as P5 and P6, respectively. (B, C) Quality control and normalization of shRNA libraries. Relative log expression (RLE) plot examines the result of the normalization method (C). Principal component analysis (PCA) plot of six shRNA libraries after removing batch effect. (D, E) Heatmaps of genes targeted by significantly enriched or depleted shRNAs compared to the input library. (D) Comparison between uninfected and input library with a threshold of a *P* value of 0.001 and a log_2_(fold change) (log_2_FC) of 3.5. (E) Comparison between the infected library and input library with a threshold of a *P* of value 0.001 and a log_2_FC of 3. Download FIG S1, TIF file, 0.9 MB. Copyright © 2020 Stelzner et al. 2020 Stelzner et al. This content is distributed under the terms of the Creative Commons Attribution 4.0 International license.

10.1128/mBio.02250-20.8 TABLE S1 Genes targeted by shRNAs differentially enriched in “no intracellular infection” versus input sample and in “intracellular infection” versus input sample (*P* < 0.001; log_2_FC >1.5 or log_2_FC <−1.5) Download Table S1, XLSX file, 0.1 MB. Copyright © 2020 Stelzner et al. 2020 Stelzner et al. This content is distributed under the terms of the Creative Commons Attribution 4.0 International license.

We next subjected the target genes of all differentially enriched or depleted shRNAs to gene set enrichment analysis (GSEA) (Fig. 1B and C; see also Table S2 in the supplemental material). Among the pathways significantly enriched in the cell population without intracellular infection, we identified the endocytosis pathway. Other pathways required for S. aureus cytotoxicity were gonadotropin-releasing hormone (GNRH) signaling, phenylalanine metabolism, genes related to GAP junctions, homologous recombination, lysine degradation, tryptophan metabolism, butanoate metabolism, and long-term potentiation. We identified two pathways, the “one carbon pool by folate/folate biosynthesis” and the calcium signaling pathway, where shRNAs were enriched in both S. aureus invasion and cytotoxicity.

10.1128/mBio.02250-20.9 TABLE S2 Gene set enrichment analysis (GSEA) of genes identified by the shRNA screen to be implicated in S. aureus invasion or cytotoxicity Download Table S2, XLSX file, 0.01 MB. Copyright © 2020 Stelzner et al. 2020 Stelzner et al. This content is distributed under the terms of the Creative Commons Attribution 4.0 International license.

### Intracellular S. aureus bacteria induce increase in cytoplasmic Ca^2+^.

**FIG 2:**
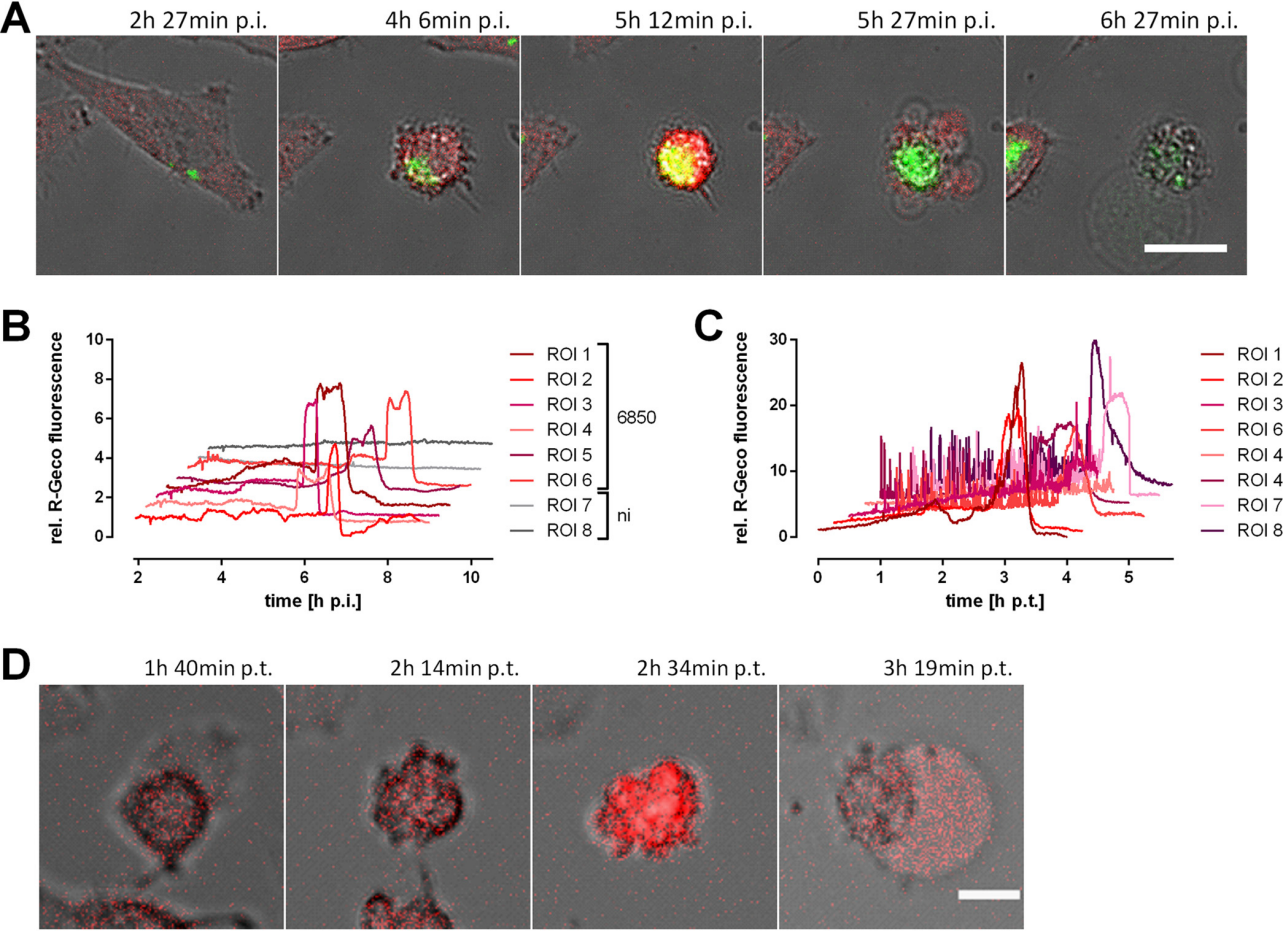
Infection with S. aureus or treatment with secreted S. aureus virulence factors leads to a massive cytosolic Ca^2+^ increase in epithelial cells. HeLa R-Geco cells were either infected with S. aureus 6850 green fluorescent protein (GFP) (A, B) or treated with 10% sterilized supernatant (SNT) of a S. aureus 6850 overnight culture (C, D) and visualized by live cell imaging. (A) Stills from time-lapse imaging are shown (green, S. aureus; red, R-Geco; gray, brightfield). Bar, 25 μm. (B) Quantification of relative R-Geco fluorescence of single cells (region of interest [ROI]) infected with S. aureus 6850 or not infected (ni) was performed over the course of infection. h p.t., hours posttransduction. (C) Relative R-Geco fluorescence of single cells was quantified upon SNT treatment. (D) Representative stills of one SNT-treated HeLa R-Geco cell (red, R-Geco; gray, brightfield). Bar, 10 μm.

**FIG 3:**
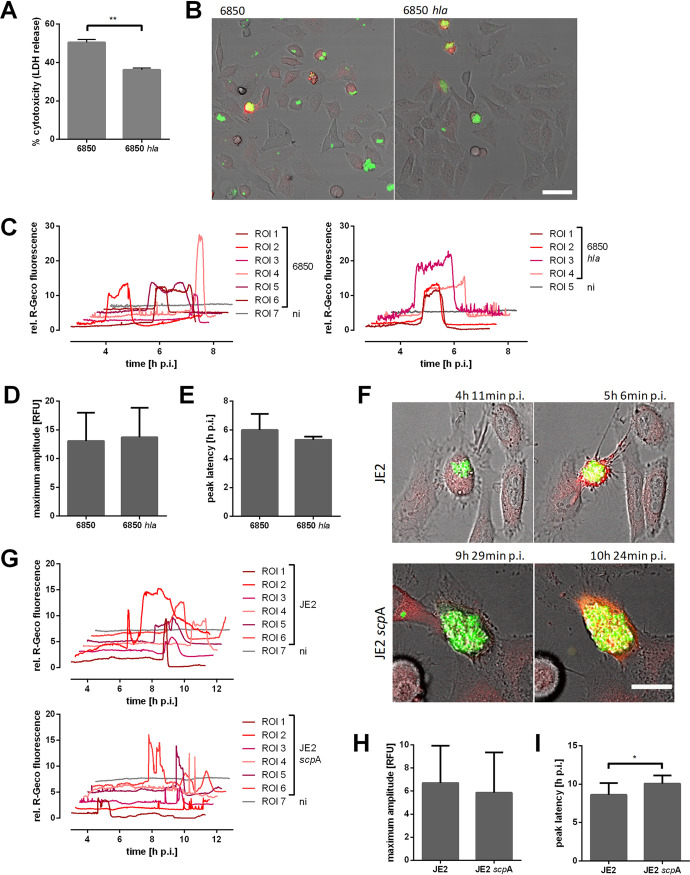
Ca^2+^ signaling in epithelial cells infected with S. aureus 6850 *hla* or JE2 *scpA* strains. (A) HeLa cells were infected with S. aureus 6850 or the 6850 *hla* mutant, and cytotoxicity was determined at 6 h p.i. by LDH release. (B to I) HeLa R-Geco cells were infected with S. aureus 6850 GFP, 6850 *hla* GFP, JE2 GFP, or JE2 *scp*A GFP strains, and cytosolic Ca^2+^ concentration, i.e., R-Geco fluorescence, was measured by time-lapse imaging. (B) Stills of time-lapse imaging at 5 h 6 min postinfection (p.i.) are shown (green, S. aureus; red, R-Geco; gray, brightfield). Bar, 50 μm. (C) Relative R-Geco fluorescence was quantified over the time period of infection in single uninfected cells (ni) or in cells infected with S. aureus 6850 or S. aureus 6850 *hla* strains. (D) The peak amplitude of the relative R-Geco fluorescence of 4 to 12 single infected cells was determined. (E) The latency of the relative R-Geco fluorescence peak after S. aureus intracellular infection was calculated in 4 to 12 single cells. (F) Representative stills from time-lapse fluorescence microscopy (green, S. aureus; red, R-Geco; gray, brightfield). Bar, 20 μm. (G) Relative quantification of cytosolic Ca^2+^ concentrations of single infected (JE2) or uninfected (ni) cells. (H) The maximum amplitude of relative R-Geco fluorescence of 10 single infected cells was determined. (I) The latency after infection until the maximum amplitude of relative R-Geco fluorescence was quantified in 10 single cells. Statistical significance was determined by unpaired *t* test (*, *P* < 0.05).

The GSEA analysis revealed the calcium signaling pathway to be involved in both S. aureus invasion and cytotoxicity. In addition, pathways connected to calcium signaling like long-term potentiation, GNRH signaling, and gap junctions, were enriched for the S. aureus intracellular cytotoxicity gene set. Long-term potentiation implies ER calcium channels and Ca^2+^-sensing and -regulated proteins, GNRH signaling is known to increase intracellular Ca^2+^ levels and PKC activation (48), and gap junctions form intercellular connections that are permeable for, e.g., calcium ions. Therefore, we investigated if intracellular S. aureus interfered with host cell Ca^2+^ signaling and thus may activate signaling pathways and Ca^2+^-sensing proteins implicated in infection-induced host cell death. We used the genetically encoded fluorescent calcium sensor R-Geco (49) and generated a transgenic HeLa cell line in order to monitor changes in cellular Ca^2+^ concentration after infection with S. aureus by time-lapse microscopy. Extracellular bacteria were removed by treatment with lysostaphin. We observed a massive increase of the cytosolic Ca^2+^ concentration after the onset of intracellular bacterial replication and host cell shrinkage, which was followed by the formation of membrane blebs and a subsequent loss of bacterial and cellular fluorescence (Fig. 2A; see also Video S1 in the supplemental material). Relative quantification of the cytosolic Ca^2+^ concentration demonstrated a roughly 2- to 6-fold increment of R-Geco fluorescence in infected cells between 5 and 7 hours postinfection (h p.i.) (Fig. 2B). The high cytosolic Ca^2+^ level persisted for several minutes and up to 1 h. No major changes in cellular Ca^2+^ concentrations were detected in uninfected cells (Fig. 2B and Video S1). This was independent of the bacterial strains used, since we observed that the methicillin-resistant and cytotoxic strain JE2 also induced a rise in cytoplasmic Ca^2+^ (Fig. 3F and G).

Since several S. aureus toxins were shown to induce Ca^2+^ flux in human cells (37), we treated HeLa R-Geco cells with 10% sterile-filtered S. aureus culture supernatant. This led to high and frequent oscillations of cytosolic Ca^2+^ in most cells, which was followed by a sustained Ca^2+^ increase (Fig. 2C and Video S2 in the supplemental material). Similarly to intracellular infection with S. aureus, this cytoplasmic Ca^2+^ overload occurred after cell contraction but prior to the formation of membrane blebs (Fig. 2D), supporting the interpretation that a secreted virulence factor may trigger Ca^2+^ fluxes from within the host cell.

The S. aureus pore-forming toxin alpha-hemolysin (*hla*) is known to form Ca^2+^-permissive pores and thus cytosolic Ca^2+^ elevations in different types of host cells (50, 51). Additionally, alpha-toxin plays a role in intracellular cytotoxicity of S. aureus (Fig. 3A) (28, 52). Therefore, we investigated the Ca^2+^ flux-inducing properties of an isogenic S. aureus *hla* deletion mutant (6850 *hla*). Interestingly, infection of HeLa R-Geco cells with the 6850 *hla* mutant did not abolish the cytosolic Ca^2+^ increase (Fig. 3B and C). Furthermore, the amplitude, as well as the latency, of maximal R-Geco fluorescence was not significantly different in HeLa cells infected with S. aureus 6850 *hla* compared to that in the wild type (Fig. 3D and E). Another virulence factor, which we recently identified as playing a role in S. aureus intracellular cytotoxicity, is the cysteine protease staphopain A (*scpA*) (32). Mutation of *scpA* in the JE2 strain also did not impair cytoplasmic Ca^2+^ increase (Fig. 3F to H); however, the onset of the cytosolic Ca^2+^ rise was delayed by 1.5 h on average (Fig. 3I) and was shorter. Furthermore, we observed Ca^2+^ oscillations (Fig. 3G). This is in concordance with previous findings, in that knockout of *scpA* delays S. aureus intracellular cytotoxicity (32).

### Infection with intracellular S. aureus causes influx of extracellular Ca^2+^ in the host cell.

**FIG 4:**
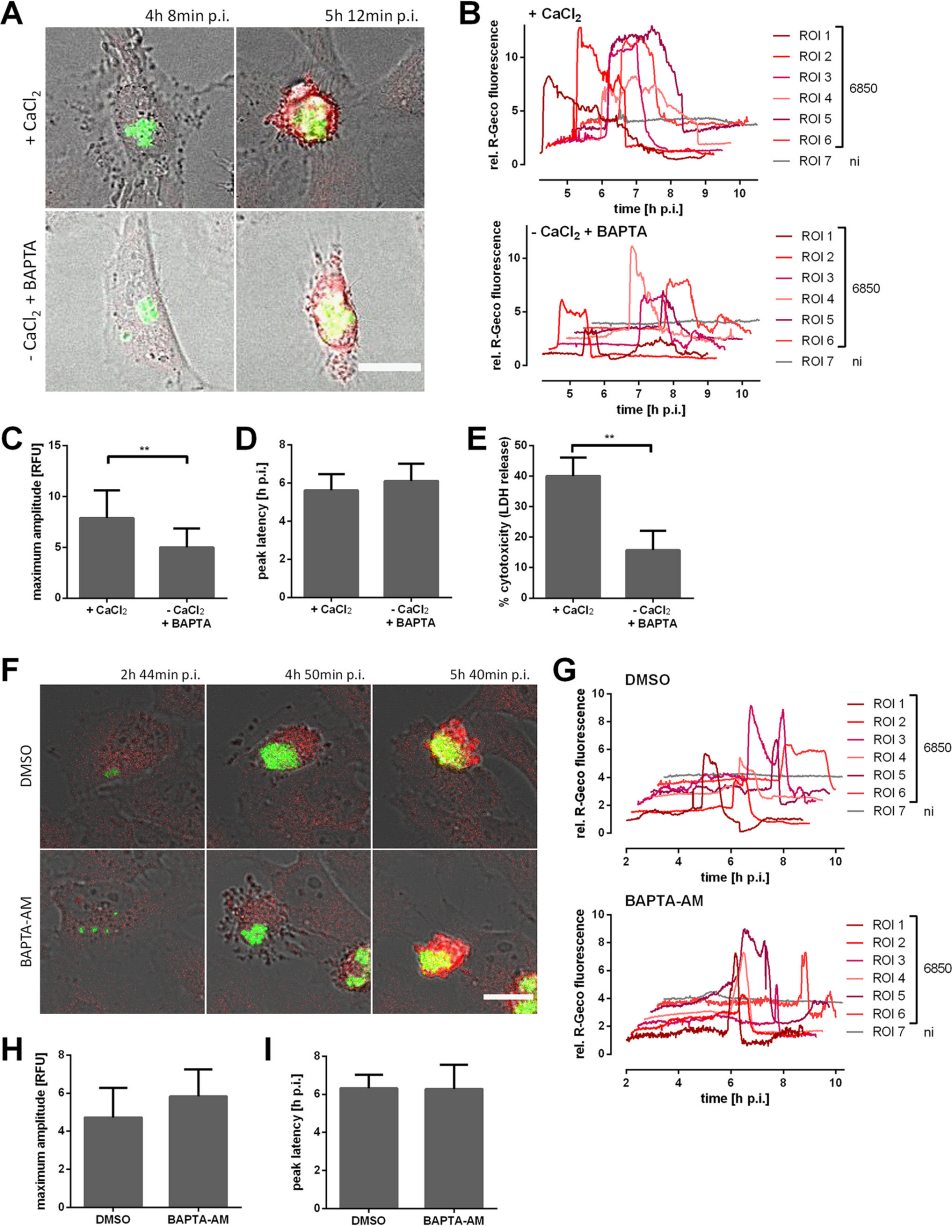
Removal of extracellular Ca^2+^ during S. aureus intracellular infection impairs cytosolic Ca^2+^ increase. (A to D) HeLa R-Geco cells were infected with S. aureus 6850 GFP, and at 3 h p.i., medium with 1.8 mM CaCl_2_ (+CaCl_2_) or without CaCl_2_ and with 0.2 mM BAPTA [1,2-bis(2-aminophenoxy)ethane-*N*,*N*,*N*′,*N*′-tetraacetic acid] (−CaCl_2_ +BAPTA) was added. Subsequently, R-Geco fluorescence was monitored by live cell imaging. (A) Representative stills of infected HeLa R-Geco cells with or without extracellular Ca^2+^ (green, S. aureus; red, R-Geco; gray, brightfield). Bar, 20 μm. (B) Relative R-Geco fluorescence of single infected (6850) or uninfected (ni) cells was quantified over the time course of infection under the different conditions. (C) The maximal amplitude of the relative R-Geco fluorescence of 6 to 11 single infected cells under the different conditions was determined. (D) The latency of the maximal relative R-Geco fluorescence peak after S. aureus intracellular infection was determined in 6 to 11 single infected cells. (E) HeLa cells were infected with S. aureus 6850, and at 3 h p.i., medium with 1.8 mM CaCl_2_ (+CaCl_2_) or without CaCl_2_ and with 0.2 mM BAPTA (−CaCl_2_ +BAPTA) was added. LDH release was quantified at 6 h p.i. (F to I) HeLa cells were infected with S. aureus 6850 GFP; at 2 h p.i., dimethyl sulfoxide (DMSO) or 5 μM BAPTA-AM [1,2-bis(2-aminophenoxy)ethane-*N*,*N*,*N*′,*N*′-tetraacetic acid tetrakis(acetoxymethyl ester)] were added, and R-Geco fluorescence was monitored over time. (F) Stills of representative infected cells are depicted (green: S. aureus; red, R-Geco; gray, brightfield). Bar, 20 μm. (G) Relative R-Geco fluorescence of single infected (6850) or uninfected (ni) cells was quantified over the time course of infection under the different conditions. (H) The maximum amplitude of the relative R-Geco fluorescence of 6 to 11 single infected cells under the different conditions was determined. (I) The latency of the relative R-Geco fluorescence peak after S. aureus intracellular infection in 6 to 11 single infected cells was calculated. Statistical analysis was performed by unpaired *t* test (**, *P* < 0.01; ***, *P* < 0.001).

We next studied the origin of the calcium ions. A preliminary test showed that a combination of Ca^2+^ withdrawal and chelation with BAPTA [1,2-bis(2-aminophenoxy)ethane-*N*,*N*,*N*′,*N*′-tetraacetic acid] resulted in a strong reduction of R-Geco fluorescence upon addition of the calcium ionophore ionomycin (see Fig. S2A in the supplemental material). Thus, HeLa R-Geco cells were infected with S. aureus 6850, and the cell culture medium was replaced with Ca^2+^-containing or Ca^2+^-free medium (including BAPTA) after bacterial invasion and phagosomal escape (3 h p.i.). Subsequently, infected cells were monitored for changes in Ca^2+^ concentration (Fig. 4A and B). When Ca^2+^ was omitted from the medium and additionally chelated with BAPTA, the relative R-Geco fluorescence showed a significantly reduced maximal amplitude (*P = *0.0004) in S. aureus-infected cells (Fig. 4C), whereas no temporal changes of the cytosolic Ca^2+^ peak were noticed upon omission of extracellular Ca^2+^ (Fig. 4D). Differences in cell morphology were not observed under the tested conditions. Additionally, Ca^2+^ withdrawal from the medium significantly reduced S. aureus intracellular cytotoxicity (*P = *0.0085) by 61% (Fig. 4E).

10.1128/mBio.02250-20.4 FIG S2 Ca^2+^ flux via plasma and ER membrane in S. aureus-induced cytoplasmic Ca^2+^ rise. (A) HeLa R-Geco cells were cultivated in medium with 1.8 mM CaCl_2_, without CaCl_2_ or without CaCl_2_ and with 0.2 mM BAPTA [1,2-bis(2-aminophenoxy)ethane-*N*,*N*,*N*′,*N*′-tetraacetic acid] and 0.5 μM ionomycin added while recording R-Geco fluorescence. (B) HeLa R-Geco cells were treated with dimethyl sulfoxide (DMSO) or 5, 10, 25, or 50 μM BAPTA-AM [1,2-bis(2-aminophenoxy)ethane-*N*,*N*,*N*′,*N*′-tetraacetic acid tetrakis(acetoxymethyl ester)], and while recording R-Geco fluorescence, 0.5 μM ionomycin was added. (C) Fluorescence images of HeLa ER-LAR-Geco cell lines (BF, brightfield; bar, 20 μm). (D) HeLa ER-LAR-Geco G-Geco cells were infected with S. aureus 6850 Cerulean, and changes in ER and cytosolic Ca^2+^ concentration were monitored by live cell imaging. Relative quantification of Ca^2+^ concentrations in cytosol and ER of single infected cells and an uninfected cell (lower right graph) are shown. (E) Fluorescence of HeLa ER-LAR-Geco G-Geco cells was recorded by live cell imaging, while 10 μM ionomycin (left) or 10 μM thapsigargin (right) was added (see arrows). Download FIG S2, TIF file, 0.3 MB. Copyright © 2020 Stelzner et al. 2020 Stelzner et al. This content is distributed under the terms of the Creative Commons Attribution 4.0 International license.

We next depleted intracellular Ca^2+^ with the cell-permeable chelator BAPTA-AM [1,2-bis(2-aminophenoxy)ethane-*N*,*N*,*N*′,*N*′-tetraacetic acid tetrakis(acetoxymethyl ester)]. Increasing concentrations of BAPTA-AM delayed, but did not reduce, an ionomycin-induced cytosolic Ca^2+^ increase (Fig. S2B). Furthermore, concentrations greater than 10 μM BAPTA-AM were cytotoxic to HeLa cells after several hours of incubation (data not shown). Therefore, we used 5 μM BAPTA-AM to treat HeLa R-Geco cells 2 h after S. aureus infection, and R-Geco fluorescence was recorded over time. However, no differences in the cytoplasmic Ca^2+^ increase between BAPTA-AM-treated and dimethyl sulfoxide (DMSO)-treated cells were observed (Fig. 4F and G). Also, amplitude and latency of the maximal Ca^2+^ concentration were not significantly altered by BAPTA-AM treatment (Fig. 4H and I). Thus, the Ca^2+^-chelating effect of BAPTA-AM was likely too small to interfere with S. aureus-induced cytosolic Ca^2+^ increase.

### Intracellular S. aureus perturbs ER Ca^2+^ homeostasis.

**FIG 5:**
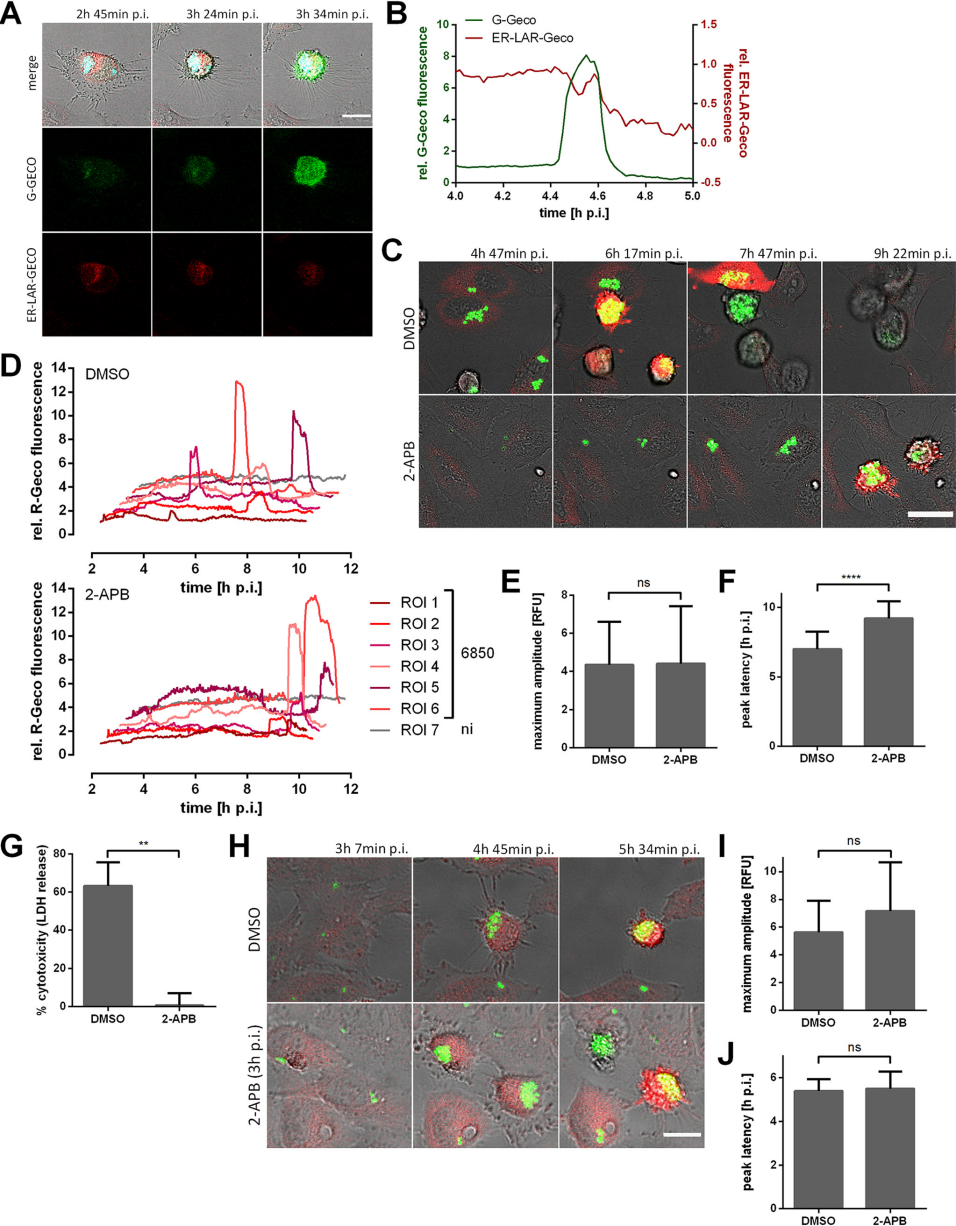
Intracellular S. aureus modulates endoplasmic reticulum (ER) Ca^2+^ concentration. (A, B) HeLa ER-LAR-Geco G-Geco cells were infected with S. aureus 6850 Cerulean, and changes in ER and cytosolic Ca^2+^ concentration were monitored by live cell imaging. (A) Representative stills from time-lapse fluorescence microscopy (cyan, S. aureus; red, ER-LAR-Geco; green, G-Geco; gray, brightfield). Bar, 20 μm. (B) Relative quantification of Ca^2+^ concentrations in the cytosol and ER of a single infected cell. (C to F) HeLa R-Geco cells were treated with 30 μM 2-aminoethyl diphenylborinate (2-APB) or DMSO as a solvent control 1 h prior to infection with S. aureus 6850 GFP, and R-Geco fluorescence was measured by time-lapse imaging. (C) Representative stills from time-lapse fluorescence microscopy (green, S. aureus; red, R-Geco; gray, brightfield). Bar, 30 μm. (D) Relative R-Geco fluorescence of single infected (6850) or uninfected (ni) cells was quantified over the time course of infection under the different conditions. (E) The peak amplitude of the relative R-Geco fluorescence of 27 to 32 single infected cells was quantified upon DMSO or 30 μM 2-APB treatment. (F) The latency of the relative R-Geco fluorescence peak after S. aureus intracellular infection was determined, and the mean value of 27 to 32 cells was calculated. (G) HeLa cells were treated with 30 μM 2-APB or DMSO as a solvent control 1 h prior to infection with S. aureus 6850, and cell death was determined at 6 h p.i. by LDH quantification. (H to J) HeLa R-Geco cells were infected with S. aureus 6850 GFP, and R-Geco fluorescence was measured by time-lapse imaging. At 3 h 7 min p.i., DMSO or 30 μM 2-APB were added. (H) Representative stills from time-lapse fluorescence microscopy (green, S. aureus; red, R-Geco; gray, brightfield). Bar, 20 μm. (I) The peak amplitude of the relative R-Geco fluorescence of 7 to 11 single infected cells was quantified after DMSO or 30 μM 2-APB treatment. (J) The latency of the relative R-Geco fluorescence peak after S. aureus intracellular infection was determined, and the mean value of 7 to 11 cells was calculated. Statistical analysis was performed by unpaired *t* test (**, *P* < 0.01; ****, *P* < 0.0001).

Since Ca^2+^ release from intracellular stores of the host cell may also contribute to the cytoplasmic Ca^2+^ increase triggered by intracellular S. aureus, we generated a reporter cell line stably expressing a red fluorescent endoplasmic reticulum (ER)-targeted fluorescent Ca^2+^ sensor (ER-LAR-Geco) (53), in addition to a green fluorescent cytosolic Ca^2+^ indicator (G-Geco). The localization of ER-LAR-Geco was compartment specific, with a typical distribution of the fluorophores to the ER (Fig. S2C). We infected HeLa reporter cells with S. aureus 6850 and monitored fluorescence over time. In contrast to the cytoplasm, only small changes in ER Ca^2+^ concentration were observed in infected host cells (Fig. 5A and B and Fig. S2D). Quantification of ER-LAR-Geco fluorescence disclosed a decrease in ER Ca^2+^ concentration concomitant with the onset of cytosolic Ca^2+^ rise, whereas ER-LAR-Geco fluorescence increased minutes later, then dropped again with the decrease of G-Geco fluorescence. Compared to the changes in cytosolic Ca^2+^ concentration, the shift of the ER-LAR Geco fluorescence was minor. To determine the dynamic range of the ER Ca^2+^ indicator, we treated HeLa ER-LAR-Geco G-Geco cells with Ca^2+^-perturbing agents. Ionomycin and thapsigargin also induced only small changes in ER-LAR-Geco fluorescence compared to G-Geco fluorescence (Fig. S2E). These results show that the ER Ca^2+^ indicator was functional and that even high concentrations of Ca^2+^-perturbing agents only trigger a small decrease in ER-LAR-Geco fluorescence.

Our observation of a simultaneous decrease in ER Ca^2+^ concentration and increase in cytosolic Ca^2+^ levels (Fig. 5B and Fig. S2D) thus suggest that store-operated calcium entry (SOCE) may be implicated in S. aureus-induced cell death. We therefore blocked SOCE with 2-aminoethyl diphenylborinate (2-APB). HeLa R-Geco cells were treated with 30 μM 2-APB 1 h before infection and were subsequently infected with S. aureus 6850 in the presence of the inhibitor. Cytosolic Ca^2+^ concentrations were monitored over the time course of infection. We found that 2-APB did not interfere with the amplitude of the bacterially induced cytoplasmic Ca^2+^ increase, but the inhibitor significantly (*P* < 0.0001) delayed the S. aureus-triggered cytoplasmic Ca^2+^ rise (Fig. 5C to F). Quantification of lactate dehydrogenase (LDH) release revealed that 2-APB treatment significantly (*P = *0.0015) reduced the cytotoxicity of S. aureus at 6 h p.i. by 99% compared to that in DMSO-treated cells (Fig. 5G). This effect of 2-APB was reproducible in other epithelial cell lines, such as A549 and 16HBE14o^−^ cells, infected with S. aureus 6850 (see Fig. S3A and B in the supplemental material). Similarly, the cytotoxicity of S. aureus JE2 in HeLa cells was abrogated by 2-APB treatment (Fig. S3C).

10.1128/mBio.02250-20.5 FIG S3 2-Aminoethyl diphenylborinate (2-APB) attenuates bacterial cytotoxicity, but not S. aureus-induced cytoplasmic Ca^2+^ increase. (A to G) A549 (A), 16HBE14o^−^ (B), HeLa (C, D, F, G), or HeLa YFP-CWT (E) cells were treated with 30 μM 2-APB or DMSO 1 h prior to infection with S. aureus 6850 (A, B), JE2 (C), 6850 GFP (D, E, G), or 6850 mRFP (F) strains. (A to C) LDH release was quantified at 6 h p.i. (D) The number of infected host cells, i.e., GFP-positive cells, was measured at 1 h p.i. (E) Translocation of bacteria into the host cell cytosol was determined by quantification of colocalization of intracellular bacteria and the cytosolic escape marker YFP-CWT at 4 h p.i. (*n* = 2). (F) The relative number of intracellular bacteria was identified by measuring the mean fluorescence intensity of infected cells at 1 and 3 h p.i. (G) Optical density at 600 nm (OD_600_) of S. aureus 6850 treated with 30 μM 2-APB or DMSO in RPMI medium without fetal bovine serum (FBS) was determined for 17.5 h at 10 min-intervals. (H) S. aureus 6850 was grown in the presence of 30 μM 2-APB or DMSO in RPMI medium without FBS and at 1.5 h, (exp, exponential growth phase) and 6 h (stat, stationary growth phase) bacteria were harvested to determine expression of alpha-toxin (*hla*) and RNAIII. Statistical analysis was performed by unpaired *t* test (*, *P* < 0.05). Download FIG S3, TIF file, 0.1 MB. Copyright © 2020 Stelzner et al. 2020 Stelzner et al. This content is distributed under the terms of the Creative Commons Attribution 4.0 International license.

Host cell invasion and phagosomal escape of S. aureus are prerequisites of intracellular cytotoxicity. We found that both were not significantly affected by 2-APB treatment (Fig. S3D to F), although we noticed a small, nonsignificant difference in intracellular bacteria at 3 h p.i. in 2-APB-treated samples compared to that in cells treated with the solvent control (Fig. S3F). Live cell imaging also showed attenuated intracellular replication of S. aureus upon 2-APB treatment (Fig. 5C). In cell culture medium, 30 μM 2-APB had only a mild inhibitory effect on S. aureus replication (Fig. S3G) and did not affect expression of virulence factors, such as alpha-toxin and RNAIII (Fig. S3H).

To minimize effects on bacterial intracellular replication, HeLa R-Geco cells were infected with S. aureus, and 2-APB was only added to infected cells at 3 h p.i. (Fig. 5H to J). Time-lapse imaging showed that intracellular S. aureus was replicating despite 2-APB treatment (Fig. 5H). However, addition of the inhibitor did not abolish the S. aureus-triggered cytosolic Ca^2+^ increase. Both amplitude and latency of the cytosolic Ca^2+^ increase were not significantly altered by 2-APB treatment under these conditions (Fig. 5I and J).

### Intracellular S. aureus induces a mitochondrial Ca^2+^ increase and cell lysis.

**FIG 6:**
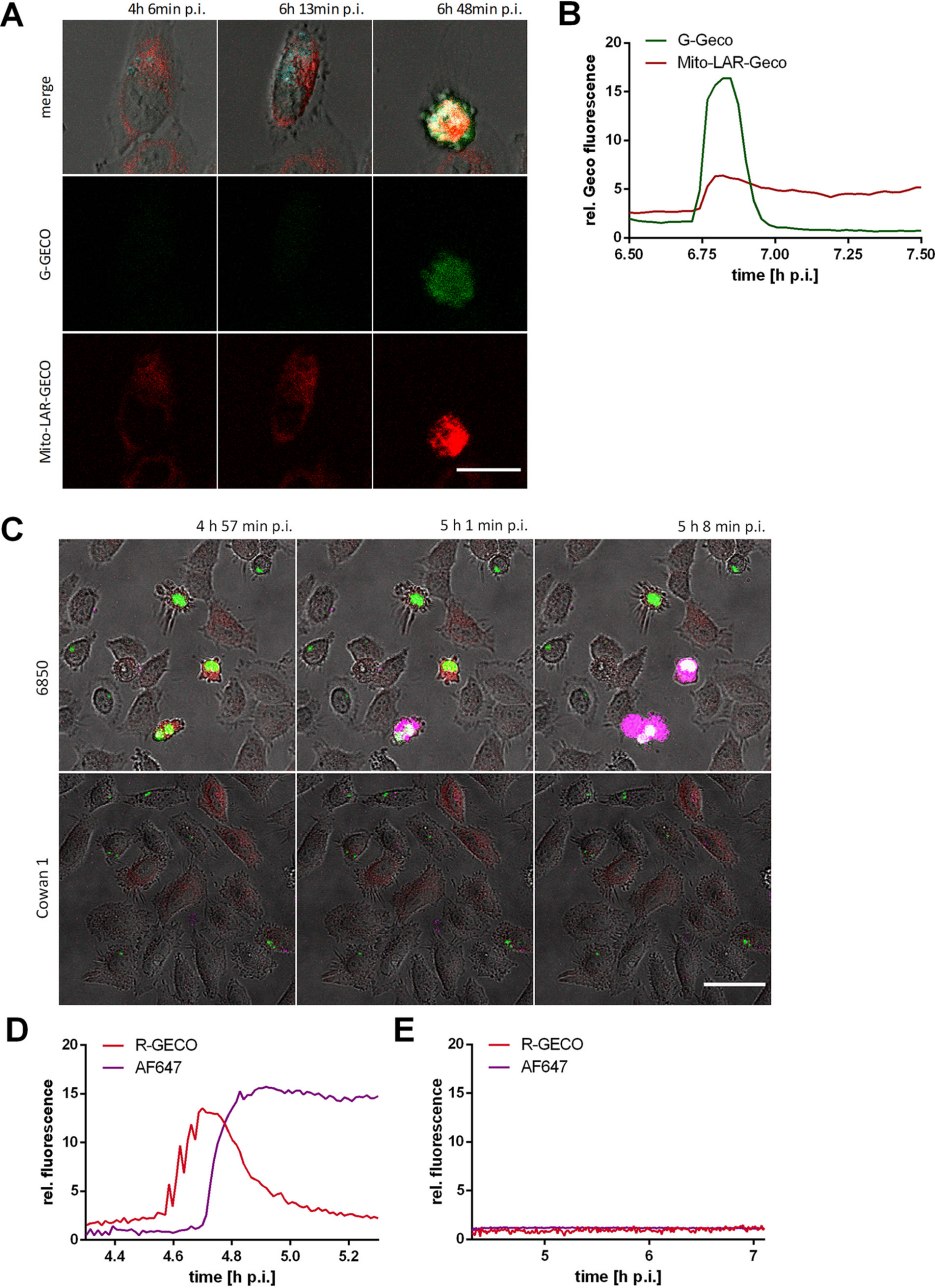
The S. aureus-triggered cytosolic Ca^2+^ increase leads to a rise of mitochondrial Ca^2+^ concentration and cell lysis. (A, B) HeLa Mito-LAR-Geco G-Geco cells were infected with S. aureus 6850 Cerulean, and changes in mitochondrial and cytosolic Ca^2+^ concentration were monitored by live cell imaging. (A) Representative stills from time-lapse fluorescence microscopy (cyan, S. aureus; red, Mito-LAR-Geco; green, G-Geco; gray, brightfield). Bar, 25 μm. (B) Relative quantification of relative Ca^2+^ concentrations in cytosol and mitochondria of a single infected cell. (C to E) HeLa R-Geco cells were infected with S. aureus 6850 GFP or Cowan 1 GFP strains, and live cell imaging was performed after addition of the fluorescent dye AlexaFluor647 hydrazide. (C) Stills from time-lapse imaging (green, S. aureus; red, R-Geco; magenta, AF64; gray, brightfield). Bar, 50 μm. Relative fluorescence of a single cell infected with S. aureus 6850 (D) or Cowan 1 (E) was quantified over the course of infection.

The second major intracellular Ca^2+^ store in addition to the endoplasmic reticulum (ER) is the mitochondria. To investigate changes in mitochondrial Ca^2+^ concentration during S. aureus infection, we generated a dual Ca^2+^ reporter cell line stably expressing a red fluorescent mitochondrially targeted fluorescent Ca^2+^ sensor (Mito-LAR-Geco) (53) in addition to a green fluorescent cytosolic Ca^2+^ indicator (G-Geco) (see Fig. S4A in the supplemental material), and we infected these cells with S. aureus. Within S. aureus-infected cells, we detected an increase in mitochondrial Ca^2+^ concentration, which ensued minutes after the increase in cytosolic Ca^2+^ (Fig. 6A and B and Fig. S4B). Interestingly, after the initial rise, no or only a partial decrease of mitochondrial Ca^2+^ was detected in contrast to G-Geco fluorescence.

10.1128/mBio.02250-20.6 FIG S4 The cytosolic Ca^2+^ increase in S. aureus-infected cells is followed by a mitochondrial Ca^2+^ increase, cell lysis, and effector caspase activation. (A) Fluorescence images of HeLa Mito-LAR-Geco G-Geco cell (BF, brightfield; bar, 20 μm). (B) HeLa Mito-LAR-Geco G-Geco cells were infected with S. aureus 6850 Cerulean and changes in mitochondrial and cytosolic Ca^2+^ concentration were monitored by live cell imaging. Relative Ca^2+^ concentrations in cytosol and mitochondria of single infected or uninfected (lower right graph) cells were quantified over time. (C) HeLa R-Geco cells were infected with S. aureus 6850 GFP, and live cell imaging was performed after addition of the fluorescent dye Alexa Fluor 647 hydrazide. Relative fluorescence of single cells infected with S. aureus 6850 or single uninfected cells (lower right graph) was quantified over the course of infection. (D) HeLa R-Geco cells were infected with S. aureus 6850 Cerulean, and CellEvent caspase-3/7 was added prior to live cell imaging. The relative R-Geco and CellEvent caspase 3/7 fluorescence of single uninfected (lower right graph) or S. aureus 6850-infected cells was quantified over time. Download FIG S4, TIF file, 0.9 MB. Copyright © 2020 Stelzner et al. 2020 Stelzner et al. This content is distributed under the terms of the Creative Commons Attribution 4.0 International license.

Mitochondrial Ca^2+^ overload is associated with necrotic cell death. We therefore tested for plasma membrane permeability in S. aureus-infected HeLa R-Geco cells by addition of the small membrane-impermeant fluorophore Alexa Fluor 647 hydrazide (AF647) to the culture medium. We observed that in infected cells, influx of the fluorescent dye ensued after the increase in cytosolic Ca^2+^ (Fig. 6C and Fig. S4C). Quantification of the relative fluorescence of single infected cells revealed that AF647 fluorescence signal started to increase when R-Geco fluorescence reached maximal levels (Fig. 6D). In contrast, uninfected cells or host cells infected with the noncytotoxic S. aureus strain Cowan 1 did not show evidence of AF647 influx and cytoplasmic Ca^2+^ increase (Fig. 6C and E and Fig. S4C).

### S. aureus-induced cytosolic Ca^2+^ rise is followed by caspase and calpain activation.

**FIG 7:**
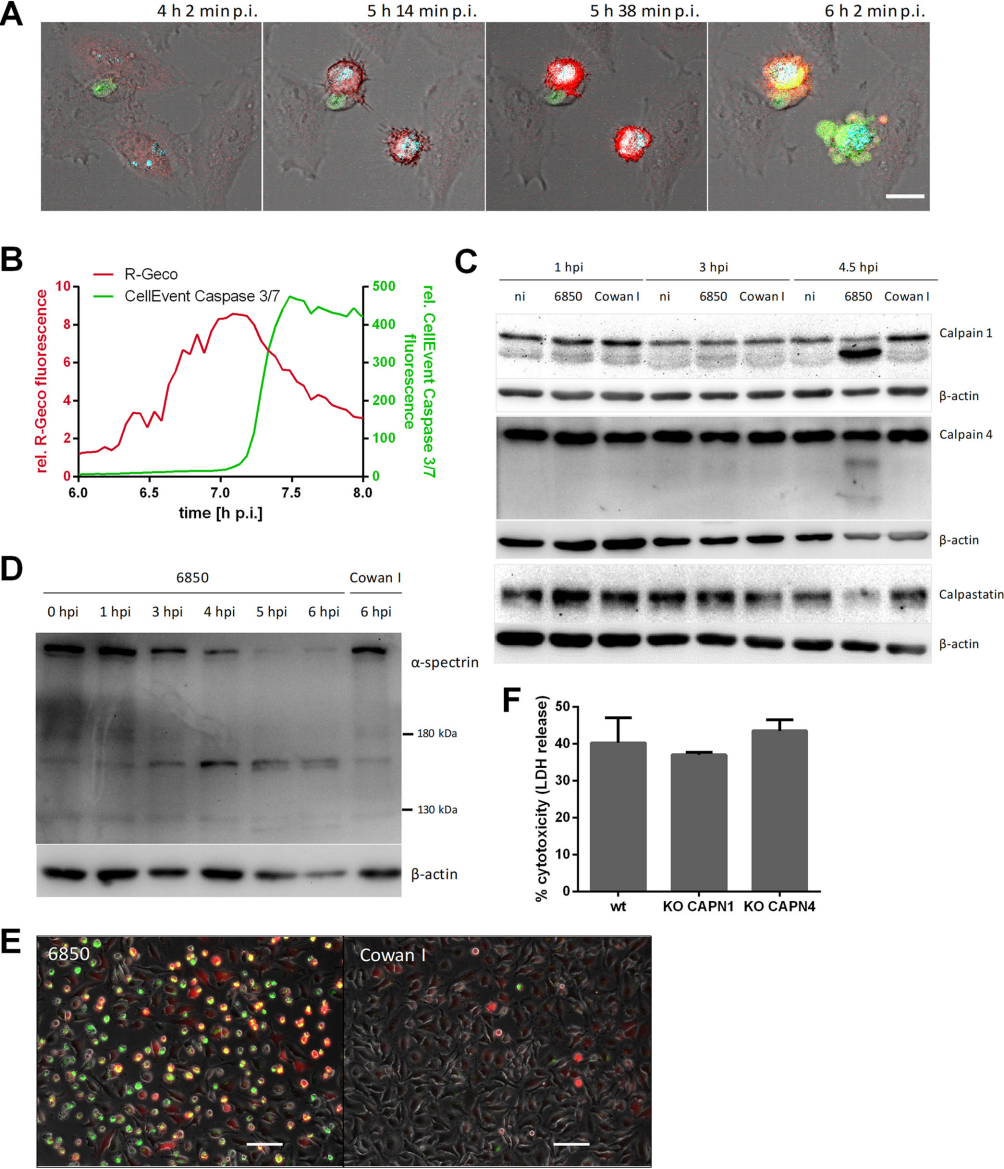
Activation of effector caspases and calpains in S. aureus-infected cells. (A, B) HeLa R-Geco cells were infected with S. aureus 6850 Cerulean and CellEvent caspase-3/7 green detection reagent was added prior to live cell imaging. (A) Representative stills from time-lapse imaging are shown (cyan, S. aureus; red, R-Geco; green, CellEvent caspase 3/7; gray, brightfield). Bar, 20 μm. (B) The relative R-Geco and CellEvent caspase 3/7 fluorescence of a single S. aureus 6850-infected cell was quantified over time. (C) HeLa cells were infected with S. aureus 6850 or the noncytotoxic strain Cowan 1 or remained uninfected (ni), and cell lysates were prepared at 1, 3, and 4.5 h p.i. Proteins were separated by SDS-PAGE and transferred onto a polyvinylidene difluoride (PVDF) membrane. Protein abundance and/or cleavage of calpain 1, calpain 4, calpastatin, and β-actin as a loading control was detected using the specific antibodies. (D) Immunoblot assay was performed as described for panel C. S. aureus 6850- or Cowan 1-infected cells were lysed at 0, 1, 3, 4, 5, and 6 h p.i. Calpain-specific cleavage of α-spectrin (150 and 145 kDa) was detected by application of an anti-α-spectrin antibody with anti-β-actin antibody as a loading control. (E) HeLa cells were loaded with a fluorogenic calpain substrate (Boc-Leu-Met-CMAC) after infection with S. aureus 6850 or Cowan 1. The presence of activated calpains was detected at 4.5 h p.i. by fluorescence microscopy (red, Boc-Leu-Met-CMAC; green, S. aureus; gray, phase contrast). Bar, 100 μm. (F) LDH release of HAP1 wild type (wt), CAPN4 knockout (KO), or CAPN1 KO cells infected with S. aureus was determined at 6 h p.i. (*n* = 2). Statistical analysis was performed by one-way analysis of variance (ANOVA).

S. aureus-induced cell death displays features of both necrosis and apoptosis (6), and Ca^2+^ is also involved in regulation of apoptosis (54). Therefore, activation of effector caspases 3 and 7 visualized by CellEvent caspase-3/7 green detection reagent was added to HeLa R-Geco cells infected with S. aureus 6850. We observed that the increase in R-Geco fluorescence was followed by an increase in CellEvent caspase 3/7 fluorescence (Fig. 7A and B). Fluorescence quantification on a single-cell level demonstrated that the effector caspases were activated in infected cells when the cytoplasmic Ca^2+^ signal peaked (Fig. 7B and Fig. S4D). In contrast, fluorescence of CellEvent caspase 3/7 and R-Geco did not increase notably in uninfected cells (Fig. S4D).

Calpains are Ca^2+^-activated cysteine proteases known to be implicated in cell death processes such as apoptosis (43). Since calpain activation involves autocatalytic proteolysis (55), we investigated the degree of calpain 4 and calpain 1 proteolysis by Western blotting at 1, 3, and 4.5 h after S. aureus infection (Fig. 7C). An increase in autocatalytically processed calpains was not observed in uninfected cells or in cells infected with the noncytotoxic control strain S. aureus Cowan 1, or well in cells infected with cytotoxic S. aureus at early time points (1 and 3 h p.i.). At 4.5 h. p.i., in contrast, calpain autolysis products were detected. At this time, we also found reduced protein levels of the calpain-specific endogenous inhibitor calpastatin (Fig. 7C). This suggested that calpains were activated in S. aureus-infected cells.

Since calpain-specific cleavage of the cytoskeletal protein α-spectrin is also used as a biomarker for calpain activation (56), we tested S. aureus 6850-infected HeLa cells by Western blotting for the calpain-specific breakdown products of α-spectrin (150 and 145 kDa). Protein bands with these molecular masses could be detected at 5 and 6 h p.i. (Fig. 7D). In contrast, infection of HeLa cells with the noncytotoxic Cowan 1 strain did not result in cleavage of α-spectrin. Additionally, a fluorogenic calpain substrate (Boc-Leu-Met-CMAC [7-amino-4-chloromethylcoumarin, t-BOC-l-leucyl-l-methioninamid]) specifically demonstrated calpain activity in host cells infected with the cytotoxic 6850 strain, but not in those infected with S. aureus Cowan 1 (Fig. 7E).

We therefore deleted the genes for calpain 1 and calpain 4 (*CAPN1* and *CAPN4*, respectively) by a CRISPR/Cas9-based approach within the haploid HAP1 cell line, in which S. aureus demonstrates an intracellular pathogenicity comparable to that in HeLa cells (see Fig. S5A to E in the supplemental material). As observed within HeLa cells, HAP1 cells demonstrated calpain activation, as well as an S. aureus-induced cytoplasmic Ca^2+^increase (Fig. S5F to H). *CAPN1* and *CAPN4* gene knockouts were verified by immunoblotting (Fig. S5I). However, the gene deletions did not reduce the cytotoxicity of S. aureus (Fig. 7F).

10.1128/mBio.02250-20.7 FIG S5 The intracellular lifestyle of S. aureus in HeLa and HAP1 cells. (A) HeLa and HAP1 cells were infected with S. aureus 6850 GFP, and invasion was determined at 1 h p.i. by flow cytometry as percentage of infected (i.e., GFP-positive) cells. (B) For detection of phagosomal escape, HeLa YFP-CWT and HAP1 YFP-CWT cells were infected with S. aureus 6850 mRFP. At 3 h p.i., phagosomal escape was determined by means of colocalization of mRFP and yellow fluorescent protein (YFP) signals. (C) For determination of intracellular replication of S. aureus 6850 GFP in HeLa and HAP1 cells, the mean GFP fluorescence (AFU), which represents the bacterial load, was measured by flow cytometry. (D) Microscopic images of uninfected and S. aureus 6850 GFP-infected HeLa and HAP1 cells at 2 and 4 h p.i. (gray, phase contrast; green, S. aureus). Bar, 50 μm. (E) HeLa and HAP1 cells infected with S. aureus 6850 were stained with the cell-impermeable dye 7-aminoactinomycin D (7-AAD) at 5 h p.i. and analyzed by flow cytometry. (F) S. aureus 6850- or Cowan 1-infected HAP1 cells were lysed at 0, 1, 3, 4, 5, and 6 h p.i. Uninfected cells or cells treated with 10 μg/ml cycloheximide (CHX) and 10 ng/ml tumor necrosis factor alpha (TNF-α) for 7 h were used as a control. Proteins were separated by SDS-PAGE and transferred onto a PVDF membrane. α-spectrin was detected using an anti-α-spectrin antibody, and an anti-β-tubulin antibody served as a loading control. (G, H) HAP1 R-Geco cells were infected with S. aureus 6850 GFP. (G) Stills of time-lapse imaging are shown (green, S. aureus; red, R-Geco; gray, brightfield). Bar, 50 μm. (H) Relative R-Geco fluorescence was quantified over the time period of infection in single uninfected cells (ni) or in cells infected with S. aureus 6850. (I) Immunoblot analysis of HAP1 CAPN1 knockout (KO) and CAPN4 KO clones generated using CRISPR/Cas9 was performed to check gene knockout. Assay was performed as described for (F), and antibodies against calpains 1 and 4 and β-actin as a loading control were used. Statistical significance was assayed using an unpaired *t* test. Download FIG S5, TIF file, 0.5 MB. Copyright © 2020 Stelzner et al. 2020 Stelzner et al. This content is distributed under the terms of the Creative Commons Attribution 4.0 International license.

## DISCUSSION

Previously conducted genome-wide screens that investigated staphylococcal virulence factors mainly involved the addition of purified toxins (57–59). However, S. aureus is readily internalized even by nonprofessional phagocytic host cells and is cytotoxic after phagosomal escape. Here, we employed a lentiviral shRNA library to identify host genes that contribute to host cell death caused by the intracellular bacteria. Host cells without intracellular infection were enriched for shRNAs targeting components of the endocytic machinery thereby supporting the hypothesis that S. aureus host cell invasion is a process driven by the host endocytosis machinery and verifying the validity of our screening procedure.

Aside from the calcium signaling pathway, the “one carbon pool by folate” pathway and “folate biosynthesis” were identified as playing a role in S. aureus invasion and intracellular cytotoxicity. Knockdown of factors involved in these pathways may result in metabolic and bioenergetic changes of the host cell and thus impair intracellular survival of S. aureus, which previously was shown to exploit the host cell carbon metabolism for intracellular replication (60, 61). Furthermore, it was shown that the intracellular bacterium is able to alter the amino acid metabolism of its host cell (61). “Phenylalanine metabolism.” “tryptophan metabolism,” and “lysine degradation” were identified by our screen to be crucial for cytotoxicity of intracellular S. aureus. Phenylalanine levels are increased in HeLa cells infected with S. aureus USA300 (60). Tryptophan metabolism is mediated by an enzyme called indoleamine 2,3-dioxygenase-1 (IDO1), which induces the conversion of tryptophan into kynurenine and downstream metabolites (62, 63). Kynurenine was shown to promote apoptosis of human and mouse neutrophils and to inhibit production of reactive oxygen species (ROS) (64). Similarly, kynurenine may be utilized by intracellular S. aureus to induce cell death in epithelial cells. The specific relationship between these pathways and intracellular infection with S. aureus remains to be elucidated.

When comparing our results to a previously published shRNA screen of S. aureus-infected cells (65), genes involved in long term potentiation were identified in both studies. Inhibition of ER Ca^2+^ refill by the SERCA inhibitor thapsigargin impaired bacterial internalization but not S. aureus intracellular replication or cytotoxicity (65). Our study, which additionally differentiated between invasion- and cytotoxicity-relevant factors, demonstrated that intracellular S. aureus induced a perturbance of intracellular Ca^2+^ homeostasis in epithelial cells that culminated in host cell death. So far, bacterial manipulation of the host cell Ca^2+^ signaling and homeostasis were only shown to be involved in the disintegration of epithelial barriers, cytoskeletal rearrangements required for cell binding or internalization of the pathogen or control of innate defense cells (66).

Secreted S. aureus virulence factors induced strong cytosolic Ca^2+^ oscillations and elevations when added extracellularly to HeLa cells (Fig. 2C). We observed that a cytosolic Ca^2+^ overload occurred after cell contraction but before the formation of plasma membrane blebs, which morphologically resembled those of intracellular S. aureus infection (compare Fig. 2A and D). Thus, virulence factors secreted by intracellular S. aureus may trigger the Ca^2+^ overload when released into the cytoplasm of the host cell. Although the inner leaflet of the plasma membrane is composed of different proteins and phospholipids compared to the outer leaflet, our results suggest that receptor-independent pore formation of S. aureus toxins may still occur from the inner side of the plasma membrane. Alpha-hemolysin, for instance, has been shown to nonspecifically integrate into membranes at high concentrations and thereby induce Ca^2+^-permissive pores (50, 51, 67–71). However, infection of epithelial cells with an alpha-toxin mutant did not significantly reduce or delay the cytosolic Ca^2+^ overload (Fig. 3B to E), suggesting that other factors are responsible for the observed phenotype. Furthermore, inactivation of the S. aureus cysteine protease staphopain A, which plays a role in S. aureus intracellular cytotoxicity (32), did not abolish cytosolic Ca^2+^ overload (Fig. 3F to I).

We further observed that, in S. aureus-infected cells, ER Ca^2+^ levels decreased together with a cytosolic Ca^2+^ rise (Fig. 5B and Fig. S2D) and that the latter also depended on calcium ions in the culture medium (Fig. 4A to C). Removal of extracellular Ca^2+^ also attenuated S. aureus intracellular cytotoxicity (Fig. 4E). This points to a role of store-operated Ca^2+^ entry (SOCE). During SOCE, a release of Ca^2+^ from the ER triggers an influx of the ion from the extracellular space to refill the internal Ca^2+^ stores (42). In order to investigate the role of SOCE in the process of S. aureus-induced Ca^2+^ perturbations and host cell death, we applied 2-APB, which inhibits SOCE at higher concentrations (≥10 to 50 μM) (72, 73). Treatment of infected cells with 2-APB led to strong reduction of S. aureus intracellular cytotoxicity and delay of bacteria-induced cytosolic Ca^2+^ increase (Fig. 5C to G), whereas it did not impair S. aureus invasion and phagosomal escape (see Fig. S6D to F in the supplemental material). S. aureus intracellular growth was attenuated upon inhibitor treatment (Fig. 5C and Fig. S3F), which may imply that SOCE is not only required for induction of cytotoxicity but also for intracellular replication of S. aureus. 2-APB had only a minor direct effect on bacterial growth, even over extended times (Fig. S3G), and it did not affect the expression of the S. aureus major virulence factors alpha-toxin or RNAIII (Fig. S3H). When infected epithelial cells were treated with 2-APB only later during S. aureus intracellular infection (approximately 3 h p.i.), intracellular S. aureus was able to replicate (Fig. 5H) but no effect of 2-APB on cytosolic Ca^2+^ increase was observed (Fig. 5I and J). Therefore, SOCE may be involved in the S. aureus intracellular lifestyle at an early time point of infection (before 3 h p.i.) and interfere with S. aureus replication. No clear conclusion regarding the involvement of SOCE in S. aureus-induced host cell death can be drawn.

The rise in cytosolic Ca^2+^ was succeeded by increased Ca^2+^ levels in mitochondria (Fig. 6B and Fig. S7B in the supplemental material). Mitochondria are involved in Ca^2+^ compartmentalization and are able to buffer cytosolic Ca^2+^ to a certain extent. Under high cytosolic Ca^2+^ concentrations, larger amounts of Ca^2+^ can accumulate in the mitochondria (74), and excessive Ca^2+^ uptake by mitochondria may lead to mitochondrial permeability transition (MPT)-driven necrosis, which is characterized by the abrupt loss of inner mitochondrial membrane osmotic homeostasis (75, 76). Cell lysis as a characteristic of a necrotic type of cell death was detected as a consequence of prolonged high cytosolic Ca^2+^ concentrations (Fig. 6C and D). Accordingly, the cytosolic Ca^2+^ rise induced by intracellular S. aureus may lead to cell death by subsequent mitochondrial Ca^2+^ overload and MPT-driven necrosis.

Interestingly, it is speculated that the facultative intracellular pathogen Shigella induces mitochondrial Ca^2+^ overload by a sustained cytosolic Ca^2+^ increase due to plasma membrane permeabilization and thus MPT-driven necrotic cell death (77–79). However, so far, local and global Ca^2+^ fluxes were detected only during host cell invasion of *Shigella* (80–82). Calpains, which are activated by Ca^2+^ release from intracellular stores likely act as executioner proteases during for MPT-driven necrosis. Additionally, in S. aureus-infected keratinocytes, degradation of the endogenous calpain inhibitor calpastatin and reduced bacterial cytotoxicity upon calpain inhibitor treatment were observed (83).

We found that calpains were also activated in S. aureus-infected HeLa cells (Fig. 7C to E). However, knockout of calpains 1 and 4 did not abolish or reduce S. aureus-induced host cell death (Fig. 7F). The high and sustained concentration of cytosolic Ca^2+^ induced by intracellular S. aureus may induce several cell death pathways in parallel by excess stimulation of Ca^2+^-sensitive targets, such as phospholipases, proteases, and endonucleases, in the cytosol (84–86). The Ca^2+^-induced damage may also trigger necrotic cell death without involving specific signaling. In this scenario, knockout of calpains cannot restrain host cell death.

Indications for involvement of an apoptotic mode of cell death in S. aureus-infected cells were found, too. The activation of effector caspases 3/7 followed after the increase in cytosolic Ca^2+^ (Fig. 7A and B). S. aureus-infected macrophages are also killed through a pathway characterized by membrane blebbing and activation of caspases 3/7, followed by cell lysis (87). Therefore, an apoptotic mode of cell death, which readily transits to a necrotic state, such as secondary necrosis, is also likely (88).

**FIG 8:**
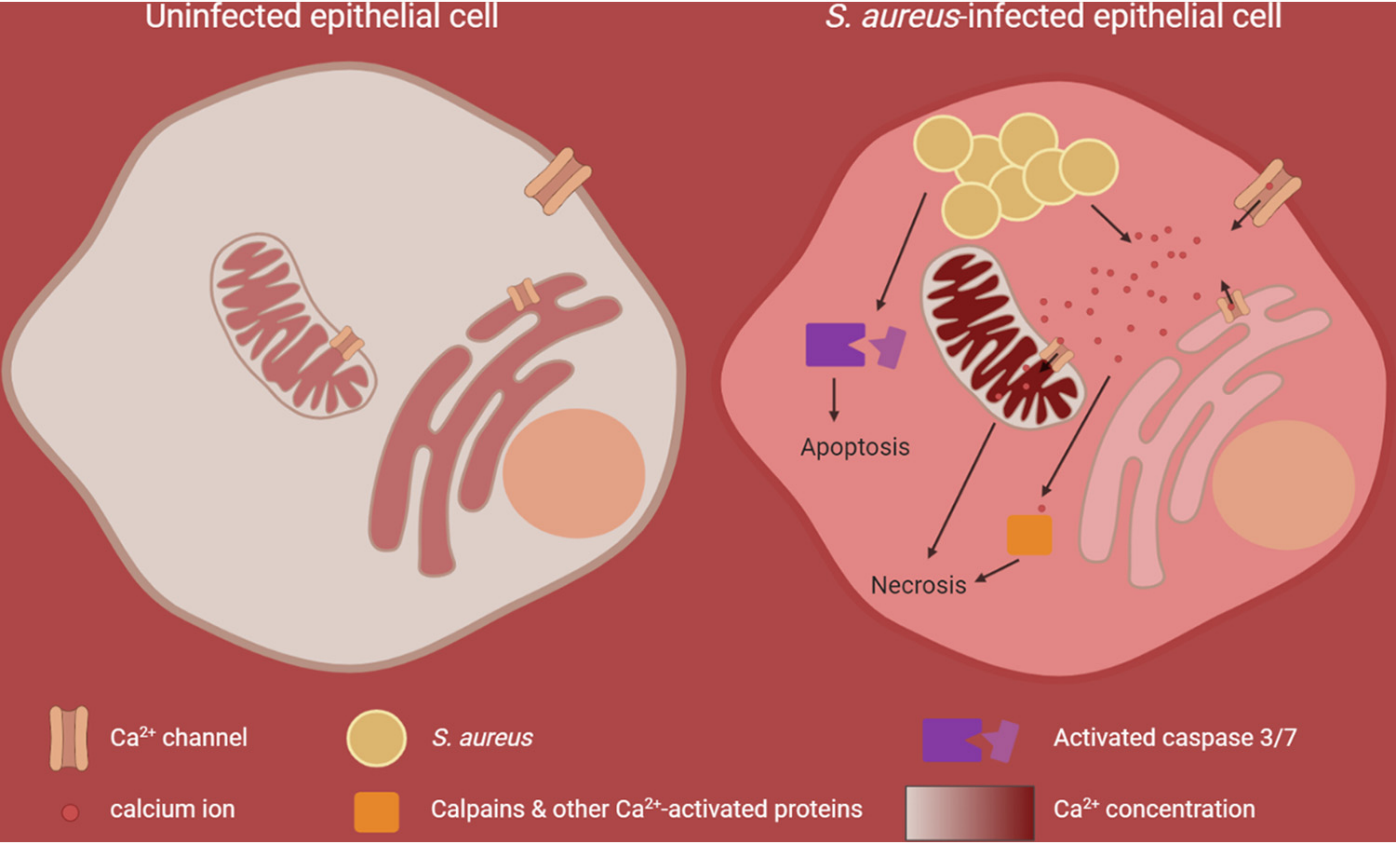
Graphical summary of S. aureus-induced perturbation of cellular Ca^2+^ homeostasis and subsequent host cell death. In uninfected epithelial cells, where the cellular Ca^2+^ homeostasis is not perturbed, the cytoplasmic Ca^2+^ concentration is 104-fold lower (approximately 100 nM) than the Ca^2+^ concentration in the extracellular milieu (approximately 1 mM). Intracellular organelles, like the endoplasmic reticulum (ER) and mitochondria, known as the internal Ca^2+^ stores, accumulate Ca^2+^ and thus exhibit a higher Ca^2+^ concentration (100 to 500 μM) compared to that of the cytoplasm. Upon S. aureus infection, calcium ions from the extracellular space and the ER are recruited to promote a cytoplasmic and subsequent mitochondrial calcium overload, which likely results in necrotic cell death. In parallel, excess stimulation of Ca^2+^-sensing proteins contributes to this effect. Additionally, effector caspases, which execute apoptotic cell death, are activated by intracellular S. aureus.

In summary, our study shows that intracellular S. aureus induces Ca^2+^ perturbations in the host cell (Fig. 8). Both plasma and ER membranes become permeable for calcium ions and induce a cytoplasmic, and subsequently a mitochondrial, Ca^2+^ overload, which results in breakdown of the plasma barrier function and cell death. Furthermore, we demonstrate that S. aureus activated calpains 1/4 and effector caspases after translocation from the endocytic compartment to the host cytosol. Several cell death pathways thus are executed in parallel by activation of multiple Ca^2+^-sensitive proteins, and we found evidence for MPT-driven necrosis as well as for apoptotic pathways. However, the damage induced by strong perturbations of the cellular Ca^2+^ homeostasis and Ca^2+^ overload may not allow reliable detection of specific signaling pathways and therefore may explain the confusing picture in the literature of host cell death pathways activated by intracellular S. aureus (6).

## MATERIALS AND METHODS

### Bacterial culture conditions.

Escherichia coli strains were grown in lysogeny broth (LB), and Staphylococcus aureus strains were grown in tryptic soy broth (TSB; Sigma), if not stated otherwise. Media were supplemented with appropriate antibiotics when necessary, and broth cultures were grown aerobically at 37°C overnight at 180 rpm. E. coli was selected on LB plates containing 100 μg/ml ampicillin, and selective TSB plates for S. aureus were prepared using 10 μg/ml chloramphenicol and/or 10 μg/ml erythromycin.

For preparation of sterile supernatant, bacteria were grown overnight at 180 rpm in TSB, and cultures were adjusted to an optical density at 600 nm (OD_600_) of 7. Subsequently, bacterial cultures were centrifuged, and the supernatant was sterile-filtered (0.22-μm pore size) and diluted to 10% with cell culture medium.

Bacterial growth curves were measured using a Tecan plate reader. Bacterial cultures were inoculated in triplicates to an OD_600_ of 0.1 in 400 μl cell culture medium without fetal bovine serum (FBS) and grown for intended periods at 37°C in a 48-well microtiter plate. Absorbance was recorded every 10 min at 600 nm.

### Generation of constructs.

All used cell lines, strains, plasmids, and oligonucleotides can be found in Table S3 in the supplemental material.

10.1128/mBio.02250-20.10 TABLE S3 Cell lines, bacterial strains, plasmids, and oligonucleotides used in this study. Download Table S3, DOCX file, 0.04 MB. Copyright © 2020 Stelzner et al. 2020 Stelzner et al. This content is distributed under the terms of the Creative Commons Attribution 4.0 International license.

In order to constitutively express the codon-adapted fluorescent reporter proteins mRFPmars and Cerulean, we amplified the SarAP1 promoter from genomic S. aureus DNA by PCR using oligonucleotides SarAP1-f and SarAP1-r. The resulting PCR product was cloned into pCR2.1-Topo (Invitrogen) and was sequence verified, restricted with SalI and KpnI, and eventually ligated into the plasmids pmRFPmars and pCerulean (89), opened with SalI and KpnI. We thereby exchanged the anhydrous tetracycline-inducible promoter, including the open reading frame (ORF), for the repressor TetR with the constitutively active SarAP1 promoter, yielding pSarAP1-mRFP and pSarAP1-Cerulean, respectively. All assembled vectors were transformed into chemically competent E. coli DH5α cells and confirmed via PCR and Sanger sequencing (SeqLab, Göttingen, Germany). Subsequently, vectors were electroporated into S. aureus RN4220 and, if required, further either electroporated or transduced (using phage φ11) into the respective strains (Table S3).

For generation of stably integrating reporter gene expression vectors, we used the lentiviral vector pLVTHM (90). Generation of the phagosomal escape reporter has been previously described (19, 91). Lentiviral vectors for expression of R-Geco, G-Geco, ER-LAR-Geco, and Mito-LAR-Geco used the following plasmids as PCR templates: CMV-R-GECO1, CMV-G-GECO1.1, CMV-ER-LAR-GECO1, and CMV-mito-LAR-GECO1.2, respectively (Table S3). The reporter genes were PCR amplified with oligonucleotides PmeI-pCMV-pLV and SpeI-BGHpolyA-pLV, thereby introducing PmeI and SpeI restriction sites. PCR products were hydrolyzed with the respective restriction enzymes (Fermentas) and were ligated into the correspondingly opened pLVTHM vector (90), thereby replacing the original enhanced green fluorescent protein (eGFP) open reading frame with the respective sensor proteins. A 20-μg aliquot of a plasmid preparation of these pLVTHM-derived plasmids was used in calcium phosphate-based cotransformation of a 15-cm dish of 293T cells along with 10 μg psPAX and 10 μg pVSVG. Dulbecco’s minimal essential medium (DMEM) growth medium was exchanged after 4 to 8 h. Two days after transfection, the supernatant was harvested and sterile-filtered (0.45-μm filter). HeLa and HAP1 cell lines were transfected with the virus particle preparations in the presence of 10 μg/ml Polybrene, and after three subculturings, the resulting transgenic cell lines were sorted on a FACSAria III cell sorter (BD).

The *hla* deletion mutant of strain 6850 was constructed using allelic replacement using the plasmid pKOR1 (92). A gene replacement cassette encompassing genomic regions upstream and downstream of the *hla* ORF was amplified by PCR with oligonucleotides attB1-*hla*-up-f and *hla*-up-r and *hla*-down-f and *hla*-down-r, respectively. Purified PCR products were restricted with SacII, ligated, and subsequently recombined into pKOR1 using BP Clonase (Invitrogen) and were subsequently transformed into competent E. coli cells. The resulting vector, pKOR1-Δ*hla*, was electroporated in S. aureus RN4220 (93), which accepts foreign DNA, and eventually in the target strain 6850. Selection of mutants was performed as described by Bae and Schneewind (92), and mutants were screened for successful gene replacement by PCR with the oligonucleotides *hla*-test-f and *hla*-test-r. Mutants were further checked for the phenotypic loss of beta-hemolysis on sheep blood agar.

### shRNA screen.

For generation of the HeLa-shRNA library, 293T cells were cultivated in five 15-cm dishes to a density of 80%. Every dish was transfected with 10 μg of pGIPZ lentiviral shRNAmir library encoding 10,000 different shRNAs, 7 μg of psPAX2, and 3.5 μg of pMD2.G using polyethylenimine (1 μl/1 μg of total DNA). Virus-containing supernatant was collected at 48 h and 72 h posttransduction and sterile-filtered, and 1 μg/μl Polybrene was added. HeLa cells cultivated in five 15-cm dishes were infected with the produced virus at a surname multiplicity of infection (MOI) of 0.1. At 24 h postinfection, medium was changed, and cells were cultivated with puromycin-containing medium for selection. These five HeLa-shRNA sublibraries encoding 10,000 different shRNAs were cultivated under puromycin pressure until they were used for infection.

One day before infection, 3 × 10^6^ HeLa-shRNA cells of each of the five subgroups were seeded in seven 15-cm dishes. Six dishes were infected with S. aureus 6850 mRFP at an MOI of 30, and 20 μg/ml lysostaphin was added 1 h p.i. in order to kill extracellular bacteria. At 4.5 h p.i., dead cells were removed by two phosphate-buffered saline (PBS) washing steps, and adherent viable cells were collected for FACS. Viable cells were selected via forward and sideward scatter (FSC-A and SSC-A) (see Fig. S1A in the supplemental material). HeLa-shRNA cells, which express green fluorescent protein (GFP), were sorted for the mRFP-positive (infection with S. aureus 6850) and mRFP-negative (uninfected) population using a FACSAria III instrument. GFP fluorescence was measured using a 488-nm laser and a 530/30-nm band pass filter for detection, and mRFP fluorescence was measured using a 561-nm laser and a 610/20-nm band pass filter. One 15-cm dish of uninfected cells served as the input control.

Genomic DNA of the sorted uninfected and infected cells and input cells was isolated with a multisource genomic DNA miniprep kit (Axygen Biosciences). The shRNA cassettes were amplified via PCR (for primers, see Table S3), and separation from the genomic DNA was obtained via agarose gel electrophoresis. Samples were diluted to a final concentration of 2 ng/μl, and Experion analysis confirmed the purity and concentration of the DNA. Samples were prepared for Illumina sequencing as suggested by the company.

High-throughput sequencing was performed with the Illumina GAIIx genome analyzer. FASTQ files were aligned with Bowtie 1 to a reference library containing the expected 10,000 shRNA sequences of each sublibrary. Alignment of the sequencing reads to the reference library was quantified. Gene features without hits in any of the libraries were eliminated prior to normalization in order to avoid bias. Data quality control and normalization were implemented in R. A batch effect among different libraries was evident by the relative log expression (RLE) plot and was carefully removed by remove unwanted variation (RUV) methods (94, 95) Further normalization and differential expression analysis were conducted with edgeR (96, 97), whereby the trimmed mean of M values (TMM) method was applied.

To expose in more detail the systematic impact of the different pathways, we further conducted a gene set enrichment analysis (98) according to the pathways of the Kyoto Encyclopedia of Genes and Genomes (KEGG) and Hallmark (gene set obtained from MSigDB ver. 7.0 [99]). We ranked all the genes with a score defined as follows: score = −log_10_(*P* value) × log_2_FC. The enrichment was computed using a permutation number of 5,000. The pathways with high positive enrichment scores together with *P* values lower than 0.02 have been carefully investigated.

### Infection of epithelial cells.

HeLa, A549, and 16HBE14o^−^ cells were grown in RPMI 1640 medium (catalog no. 72400021; Thermo Fisher Scientific), and HAP1 cells were grown in Iscove’s modified Dulbecco's medium (IMDM) (catalog no. 21980032; Thermo Fisher Scientific), each supplemented with 10% FBS (Sigma-Aldrich) and 1 mM sodium pyruvate (Thermo Fisher Scientific), at 37°C and 5% CO_2_. For infection, 0.8 × 10^5^ to 1 × 10^5^ cells were seeded into 12-well microtiter plates 24 h prior to infection. At 1 h prior to infection, medium was renewed and, if required, treatment with 30 μM 2-APB (Sigma-Aldrich) was applied.

Bacterial overnight cultures were diluted to an OD_600_ of 0.4 and incubated for 1 h at 37°C and 180 rpm to reach the exponential growth phase. Then, bacteria were washed twice by centrifugation and used to infect HeLa cells at a multiplicity of infection (MOI) of 50, if not stated otherwise. After 1 h of cocultivation, extracellular bacteria were removed by 30 min of treatment with 20 μg/ml lysostaphin (AMBI), followed by washing and further incubation in medium containing 2 μg/ml lysostaphin and, if required, 30 μM 2-APB, until the end of experiment.

### Live-cell imaging.

Phase contrast and fluorescence microscopic images of live cells were acquired with a Leica DMR microscope connected to a SPOT camera using a 10× (Leica HC PL Fluotar, numerical aperture [NA] = 0.32) or 20× (Leica C Plan, NA = 0.3) objective and VisiView software.

For time-lapse imaging, HeLa cells were seeded in 8-well chamber μ-slides (ibidi) 24 h prior to infection. Infection with fluorescent protein-expressing bacterial strains was performed at an MOI of 5, as described above. Time-lapse imaging of the samples was performed on a Leica TCS SP5 confocal microscope using a 20× (Leica HC PL APO, NA = 0.7) dry objective or 40× (Leica HC PL APO, NA = 1.3) or 63× (Leica HCX PL APO, NA = 1.3 to 0.6) oil immersion objective. Prior to imaging, cell culture medium was substituted with imaging medium (RPMI 1640 without phenol red and containing 10% FBS and 2 μg/ml lysostaphin) containing inhibitors, fluorogenic enzyme substrates, and/or chemicals, if indicated. For Ca^2+^ depleted conditions, DMEM without calcium (catalog no. 21068028; Thermo Fisher Scientific) substituted with 0.2 mM BAPTA (Merck Millipore) was used. As a positive control, 1.8 mM CaCl_2_ was added to this medium. Chelation of intracellular calcium ions was performed using BAPTA-AM (Merck Millipore). CellEvent caspase-3/7 green detection reagent (5 μM; Thermo Fisher Scientific) was applied for monitoring effector caspase activation, and 2.5 μM Alexa Fluor 647 hydrazide was applied for detecting cell lysis.

The μ-slides were transferred to a prewarmed live-cell incubation chamber surrounding the confocal microscope, and a temperature of 37°C was applied during imaging. LAS AF software was used for setting adjustment and image acquisition. All images were acquired at a resolution of 1,024 × 1,024 pixels and recorded in 8-bit mode at predefined time intervals. In certain cases, chemical treatment was performed during imaging *in situ* at the live-cell incubation chamber. All image processing steps were performed using Fiji (100). For quantification of fluorescence intensities, raw imaging data were used. For single-cell analysis, one region of interest (ROI) was defined for all recorded time frames. Fluorescence intensities (mean of relative fluorescence units [RFU]) were measured, background was subtracted, and data were normalized to time point zero (*R*_0_) to obtain relative fluorescence values. The maximum amplitude was determined as the highest relative fluorescence value measured, and peak latency was defined as the time point of maximum amplitude.

### Cytotoxicity assays.

HeLa cells were infected as described above. At the desired time point after infection, medium, which possibly contained detached dead cells, was collected from the wells, and adherent cells were detached using TrypLE (Thermo Fisher Scientific). Adherent and suspension cells of each sample were pooled, and after centrifugation for 5 min at 800 × *g*, cells were carefully resuspended in cell culture medium with 10 μl/ml 7-aminoactinomycin D (7-AAD; BD Biosciences). After 10 min of incubation in the dark, cells were immediately analyzed by flow cytometry using a FACSAria III instrument (BD Biosciences) and BD FACSDiva Software (BD Biosciences). Forward and sideward scatter (FSC-A and SSC-A) were used to identify the cell population, and doublet discrimination was performed via an FSC-H versus FSC-W and SSC-H versus SSC-W gating strategy. 7-AAD fluorescence was measured using a 561-nm laser for excitation and a 610/20-nm band pass filter for detection. In total, 10,000 events were recorded for each sample. Uninfected cells served as a negative control.

The LDH assay was performed as described previously (32). Briefly, HeLa cells were infected as described above, and at 1.5 h after infection medium was replaced by RPMI 1640 without phenol red containing 1% FBS and 2 μg/ml lysostaphin. At 6 h after infection, medium was removed from the wells, briefly centrifuged, and 100 μl of supernatant of each sample were transferred into the well of a 96-well microtiter plate in triplicates. LDH release was measured using the Cytotoxicity Detection Kit Plus (Roche) according to the manufacturer’s instruction.

### Invasion and intracellular replication assay.

HeLa cells were infected with GFP-expressing strains and prepared for flow cytometry as described above. Adherent and suspension cells were resuspended in fresh medium without phenol red containing 1% FBS and 2 μg/ml lysostaphin. To determine invasion, infected cells at 1 h p.i. were treated with 20 μg/ml lysostaphin for 10 min to remove extracellular bacteria, and the percentage of GFP-positive cells representing the infected cells was measured by flow cytometry. Intracellular replication was determined by measurement of GFP fluorescence (arbitrary units [AU]) of the infected cells at 1 and 3 h after infection by flow cytometry using a FACSAria III instrument (BD Biosciences). The intensity of GFP fluorescence corresponds to the amount of intracellular bacteria. Gating and analysis were performed as described above. GFP fluorescence was measured using a 488-nm laser and a 530/30-nm band pass filter for detection. Uninfected cells served as a negative control.

### Phagosomal escape assay.

Phagosomal escape was determined as described previously (32). HeLa YFP-CWT cells, which encode a cytoplasmic fluorophore (yellow fluorescent protein [YFP]) coupled to the staphylococcal cell wall binding domain (CWT) of lysostaphin allowing detection of cytoplasmic S. aureus, were infected with mRFP-expressing bacterial strains at an MOI of 10 in a 24-well μ-plate (ibidi). After synchronization of infection by centrifugation, infected cells were incubated for 1 h to allow bacterial invasion. Subsequently, a 30-min treatment with 20 μg/ml lysostaphin removed extracellular bacteria, after which the cells were washed and medium with 2 μg/ml lysostaphin was added. At 3 hours after infection, cells were washed, fixed with 4% paraformaldehyde overnight at 4°C, and permeabilized with 0.1% Triton X-100, and nuclei were stained with Hoechst 34580. Images were acquired with an Operetta automated microscopy system (PerkinElmer) and analyzed with the included Harmony Software. Colocalization of YFP-CWT and mRFP signals indicated phagosomal escape.

### RNA isolation.

Bacteria were grown in infection medium without FBS with 30 μM 2-APB or DMSO as a solvent control at 37°C under shaking conditions (180 rpm) for 1.5 h (exponential phase) and 6 h (stationary phase), and OD_600_ was adjusted to 0.14 or 0.46, respectively. Bacterial pellets were frozen in liquid nitrogen and stored at −80°C. RNA was extracted using the previously described TRIzol method (101). Briefly, bacterial pellets were resuspended in 400 μl buffer A (10% glucose, 12.5 mM Tris [pH 7.6], and 10 mM EDTA) and mixed with 60 μl 0.5 M EDTA. The samples were transferred to lysing matrix B tubes (MP Biomedicals) containing 500 μl Roti aquaphenol (Roth) and mechanically lysed at 6 m/sec for 45 s at 4°C using the FastPrep cell disruptor (FP120). After centrifugation, the aqueous phase was collected, mixed with 1 ml TRI reagent solution (Invitrogen), and incubated for 5 min at room temperature. Chloroform (100 μl) was added, mixed, and incubated for 3 min. After centrifugation, the aqueous phase was collected and chloroform extraction was repeated. RNA was precipitated using 500 μl isopropanol. After 15 min of incubation, the RNA pellet was washed with 75% ethanol, and the dried pellet was resuspended in diethyl pyrocarbonate water.

The Turbo DNA-*free* DNase kit (Invitrogen) was used to remove DNA contamination in RNA samples according to the manufacturer’s instructions. Briefly, 10 μg isolated RNA was mixed with DNase I in Turbo DNase buffer and incubated for 30 min at 37°C, followed by addition of a bead-based DNase inactivation reagent to stop the reaction. After 5 min of incubation time, the mixture was sedimented by centrifugation and the aqueous layer was collected. DNA digestion was verified by PCR using quantitative real-time PCR (qRT-PCR) primers against *gyrB* (Table S3).

### Quantitative real-time PCR (qRT-PCR).

Complementary DNA (cDNA) was generated using the RevertAid first-strand cDNA synthesis kit (Thermo Scientific). Briefly, 1,000 ng DNA was mixed with random hexamer primers and incubated for 5 min at 65°C. Reaction buffer, nucleotide mix, RNase inhibitor, and reverse transcriptase were added and incubated for 5 min at 25°C, followed by 60 min at 42°C and 5 min at 70°C.

Quantitative real‐time PCR was performed on a StepOne Plus PCR system (Applied Biosystems) in a 96-well plate format. The reaction mixture was composed of Green mastermix (Genaxxon), 300 nM qRT-PCR primers (Table S3), and 100 ng cDNA in a total volume of 20 μl. Reactions were run for 40 cycles at 95°C for 15 s and 60°C for 1 min after an initial holding stage of 10 min at 95°C. At the end of each run, a melting curve was performed with 0.3°C temperature increments. Analysis of relative gene expression was done according to the threshold cycle (2^−ΔΔ^*^CT^*) method (102). Gene expression was normalized to the expression levels of the housekeeping gene *gyrB*. Nontemplate controls were run to verify the lack of contamination.

### Gene knockout using CRISPR/Cas9.

For designing gene-specific single guide RNAs (sgRNAs), the CRISPOR online tool (crispor.tefor.net) was used, and gene sequences were retrieved from Ensemble (www.ensembl.org). Exons coding for essential function(s) of the protein were chosen for genetic manipulation. Synthetized sgRNA oligonucleotides (Table S3) were cloned into pSp-Cas9(BB)-2A-GFP according to the protocol from Ran et al. (103). Insertion of the sgRNA was verified by Sanger sequencing (SeqLab) using primer U6-fwd.

For plasmid transfection, HAP1 cells with a low passage number were seeded into 6-well microtiter plates at a density of 5 × 10^5^ cells/well in IMDM (catalog no. 21980032; Thermo Fisher Scientific) containing 10% FBS (Sigma-Aldrich) and antibiotic 24 h before transfection. pSp-Cas9(BB)-2A-GFP-sgRNA (3.6 μg) and pSc-TIA-CMV-BSR-TIA (400 ng) were mixed with polyethylenimine and Opti-MEM (catalog no. 51985026; Thermo Fisher Scientific) for transfection of cells. After 24 h, cells were diluted and seeded in cell culture dishes (100 × 20 mm). The next day, selection was applied by treatment with 30 μg/ml blasticidin. Subsequently, cells were monitored over 2 to 3 weeks and, if necessary, cells were washed and medium was renewed. When cell colonies were large enough, single colonies were each transferred to a well of a 12-well plate using trypsin-soaked, sterile Whatman paper. Gene knockout was verified by Western blotting.

### SDS-PAGE and immunoblotting.

Protein samples from human cells were prepared in 2× Laemmli buffer (100 mM Tris/HCl [pH 6.8], 20% glycerol, 4% SDS, 1.5% β-mercaptoethanol, and 0.004% bromophenol blue) and immediately incubated at 95°C for 10 min for protein denaturation. Proteins were separated via gel electrophoresis on 7.5 to 12% polyacrylamide gel and transferred to a polyvinylidene difluoride (PVDF) membrane (Sigma-Aldrich) using a semidry blotting system. The PVDF membrane was incubated for 1 h in blocking solution (5% human serum in 1× Tris-buffered saline with Tween 20 [TBST]) and overnight at 4°C with the first antibody (diluted in blocking solution). Antibodies against Calpain 1 (1:1,000, catalog no. 2556; Cell Signaling Technology), calpain 4 (1:500, catalog no. MAB3083; Merck Millipore), calpastatin (1:1,000, catalog no. 4146; Cell Signaling Technology), α-spectrin (1:300, catalog no. sc-48382; Santa Cruz Biotechnology), β-actin (1:3,000, catalog no. A5441; Sigma-Aldrich), and β-tubulin (1:1,000, catalog no. MAB3408; Merck Millipore) were used. Primary antibodies were detected with a horseradish peroxidase (HRP)-conjugated secondary antibody (1:3,000, catalog no. 170-6515 [Bio-Rad] or catalog no. 12-349 [Merck Millipore]) in 1× TBST with 5% nonfat dry milk using enhanced chemiluminescence (ECL) and an imaging system (Intas Science Imaging).

### Calpain activity assay.

HeLa cells were infected with S. aureus 6850 GFP as described above. At 4 h p.i., 10 μM CMAC peptidase substrate t-BOC-Leu-Met (Thermo Fisher Scientific) was added to the cells and incubated for 30 min at 37°C and 5% CO_2_. Subsequently, phase contrast and fluorescence microscopic images of live cells were acquired with a Leica DMR camera.

### Statistical analyses.

Data were analyzed using Prism ver. 6.01 (GraphPad Software). For statistical analysis, three biological replicates were performed, if not indicated otherwise. All data are presented as means with standard deviation (SD). *P* values of ≤0.05 were considered significant. Pairwise comparisons were assessed using the unpaired Student’s *t* test. Analysis of variance (ANOVA) was performed to determine whether means in a group was significantly different from each other. ANOVA was performed with Tukey’s *post hoc* analysis for defining individual differences.

10.1128/mBio.02250-20.1 VIDEO S1 Intracellular S. aureus induces a rise in cytosolic Ca^2+^. HeLa R-Geco cells were infected with S. aureus 6850 and imaged over time to visualize intracellular Ca^2+^ concentrations (green, S. aureus; red, R-Geco; gray, brightfield). Download Movie S1, MPG file, 1.1 MB. Copyright © 2020 Stelzner et al. 2020 Stelzner et al. This content is distributed under the terms of the Creative Commons Attribution 4.0 International license.

10.1128/mBio.02250-20.2 VIDEO S2 S. aureus secreted factors induce cytosolic Ca^2+^ signaling and overload. HeLa R-Geco cells were treated with 10% sterilized supernatant of an S. aureus 6850 overnight culture and imaged over time to visualize intracellular Ca^2+^ concentrations (red, R-Geco; gray, brightfield). Download Movie S2, MPG file, 1.1 MB. Copyright © 2020 Stelzner et al. 2020 Stelzner et al. This content is distributed under the terms of the Creative Commons Attribution 4.0 International license.
