# Supplementary figures and images for: Intracellular Staphylococcus aureus Perturbs the Host Cell Ca2+ Homeostasis To Promote Cell Death
Source: mBio. 2020 Dec 15;11(6):e02250-20. doi: 10.1128/mBio.02250-20 (PMC7773986; doi:10.1128/mBio.02250-20)

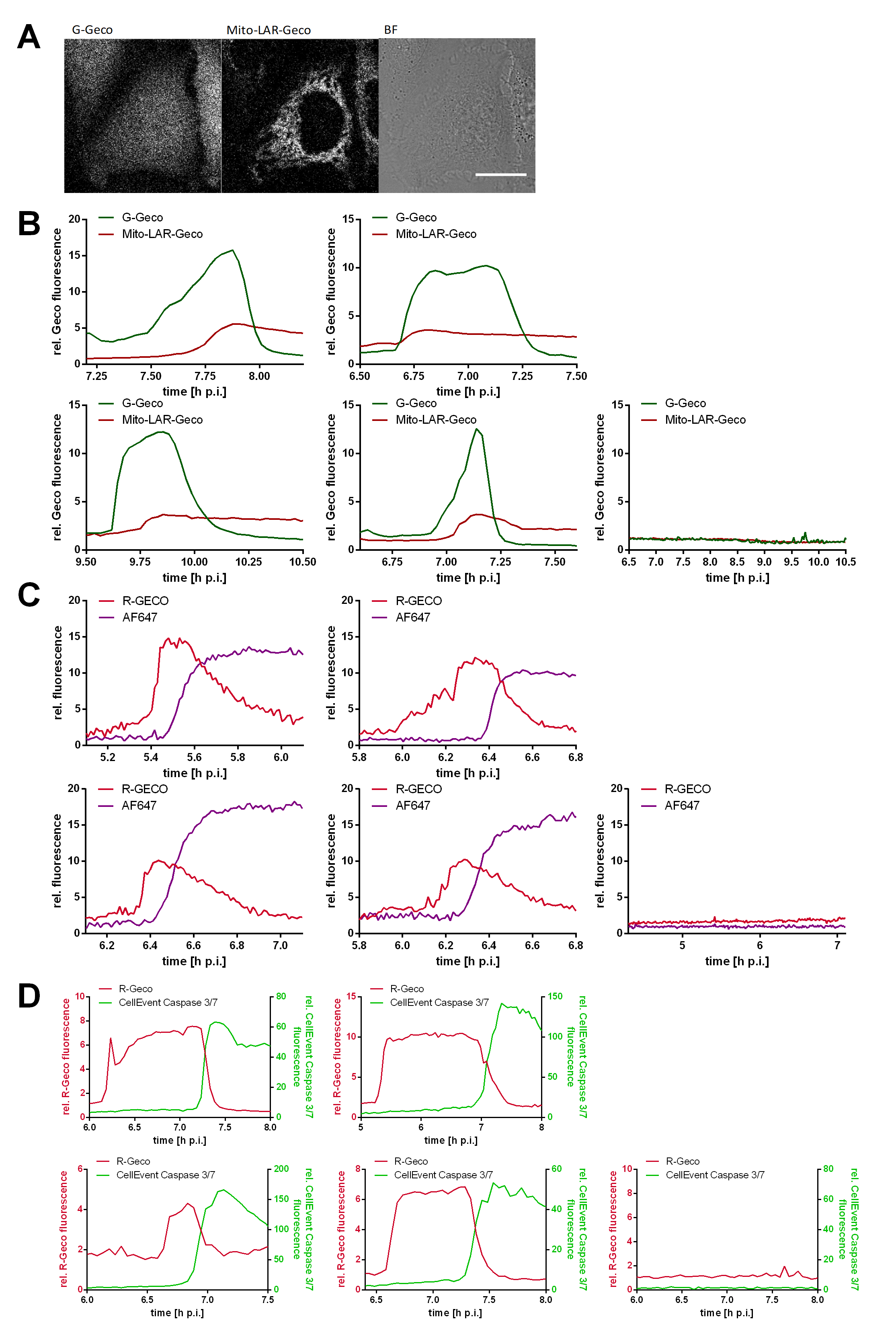

Supplement: FIG S4 [file mBio.02250-20-sf004.tif]

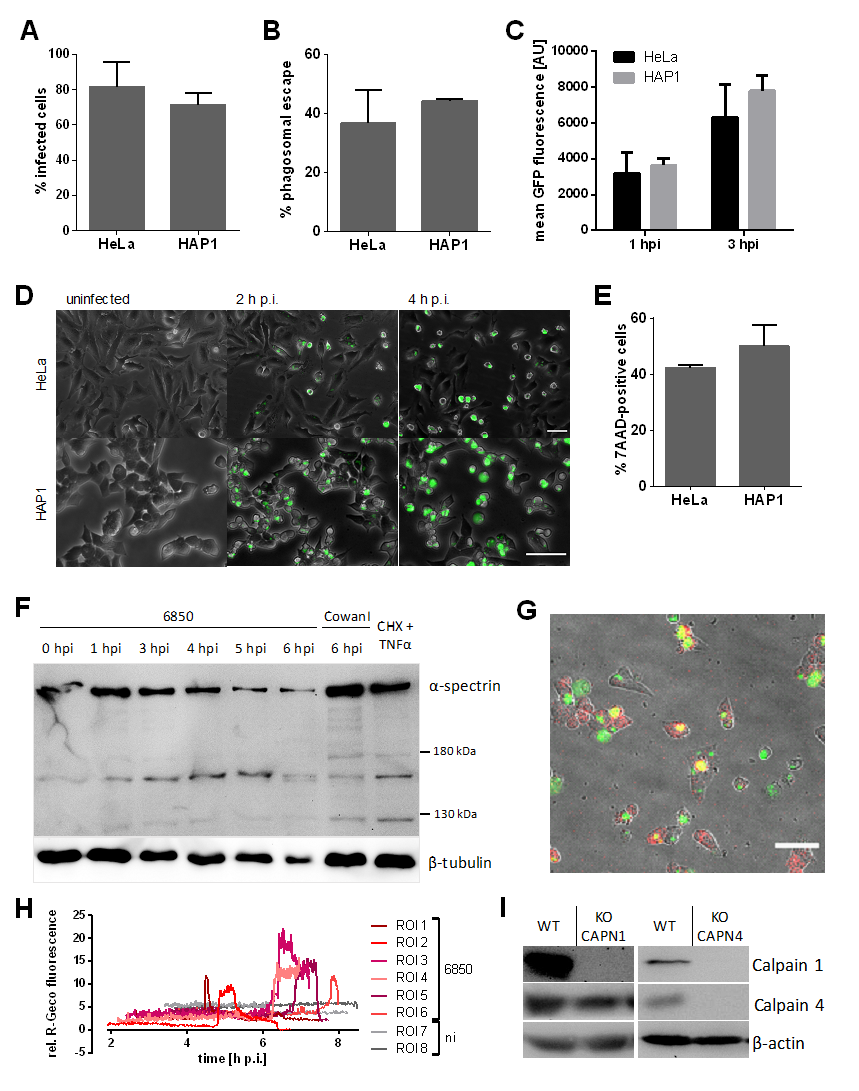

Supplement: FIG S5 [file mBio.02250-20-sf005.tif]
