# Supplementary material for: Intracellular Staphylococcus aureus Perturbs the Host Cell Ca2+ Homeostasis To Promote Cell Death
Source: mBio. 2020 Dec 15;11(6):e02250-20. doi: 10.1128/mBio.02250-20 (PMC7773986; doi:10.1128/mBio.02250-20)
Supplement: TABLE S3 [file mBio.02250-20-st003.docx]

| **Cell line** | | **Source/Reference** |
| --- | --- | --- |
| HEK 293T/17 | | ATCC CRL-11268 |
| HeLa 229 | | ATCC CCL-2.1 |
| HeLa YFP-cwt | | M. Grosz et al. (1) |
| HeLa R-Geco | | This study |
| HeLa ER-LAR-Geco G-Geco | | This study |
| HeLa Mito-LAR-Geco G-Geco | | This study |
| A549 | | D. J. Giard et al. (2) |
| 16HBE14o^-^ | | B. N. Kreiswirth et al. (3) |
| HAP1 | | J. E. Carette et al. (4) |
| HAP1 YFP-cwt | | This study |
| HAP1 R-Geco | | This study |
| HAP1 KO CAPN1 | | This study |
| HAP1 KO CAPN4 | | This study |
|  | | |
| **Bacterial strain** | **Description** | **Source/Reference** |
| ***Escherichia coli*** | | |
| DH5α | K-12 derivate, F^-^, *end*A1, *hsd*R17 (r_k_^-^, m_k_^-^), *sup*E44, *thi*-1, *rec*A1, *Gyr*A96, *rel*A1, λ^-^, Δ(*lacZYA-argF*)U169, Φ80*dlac*Z ΔM15, *deo*R, *nup*G | BRL Life Technology |
| ***Staphylococcus aureus*** | | |
| 6850 | Clinical osteomyelitis isolate, methicillin-sensitive | J. M. Vann and R. A. Proctor (5) |
| 6850 GFP | pJL74-SarAP1-GFP, expressing GFP as molecular marker | This study |
| 6850 mRFP | p SarAP1-mRFPmars, expressing mRFP as molecular marker | This study |
| 6850 Cerulean | pSarAP1-Cerulean, expressing Cerulean as molecular marker | This study |
| 6850 *hla* | *hla* deletion mutant | This study |
| 6850 *hla* GFP | pJL74-SarAP1-GFP, expressing GFP as molecular marker | This study |
| JE2 | derivative of LAC, which was cured of three plasmids, USA300 PFGE type, CA-MRSA | P. D. Fey et al. (6) |
| JE2 GFP | JE2 pGFPsf, JE2 expressing GFPsf as molecular marker | K. Stelzner et al. (7) |
| JE2 *scp*A GFP | JE2 *scp*A::*bursa* pGFPsf, JE2 *scp*A expressing GFP as molecular marker | K. Stelzner et al. (7) |
| Cowan 1 | NCTC 8530, isolated from septic arthritis, *agr* dysfunction, low expression of toxins and proteases | ATCC 12598 |
|  | | |
| **Plasmid** | **Description** | **Source/Reference** |
| **bacterial expression** | | |
| pJL74-SarAP1-GFP | vector expressing GFP, Erm^R^ | J. Liese et al. (8) |
| pSarAP1-mRFPmars | p2085-SarAP1-mRFPmars, vector expressing mRFP in *S. aureus* under control of the constitutive sarAP1 promoter, Cm^R^ | This study |
| pSarAP1-Cerulean | p2085-SarAP1-cerulean, vector expressing cerulean in *S. aureus* under control of the constitutive sarAP1 promoter, Cm^R^ | This study |
| **mammalian expression** | | |
| psPAX2 |  | Addgene (#12260), M. Wiznerowicz and D. Trono (9) |
| pMD2.G |  | Addgene (#12259), M. Wiznerowicz and D. Trono (9) |
| pLVTHM |  | Addgene (#12247), M. Wiznerowicz and D. Trono (9) |
| Open Biosystems pGIPZ shRNA library | releases 6.1–6.12 | Dharmacon; GE Lifesciences |
| YFP-cwt | cytoplasmic expression of a recruitment marker recognizing Gram-positive peptidoglycan coupled to YFP | M. Grosz et al. (1) |
| CMV-R-GECO1 | expression of red fluorescent genetically encoded Ca^2+^-indicator | Addgene (#32444), Y. Zhao et al. (10) |
| CMV-G-GECO1.1 | expression of green fluorescent genetically encoded Ca^2+^-indicator | Addgene (#32445), Y. Zhao et al. (10) |
| CMV-ER-LAR-GECO1 | expression of LAR-GECO1 in the mitochondria | Addgene (#61244), J. Wu et al. (11) |
| CMV-mito-LAR-GECO1.2 | expression of LAR-GECO1.2 in the endoplasmic reticulum | Addgene (#61245), J. Wu et al. (11) |
| pSpCas9 (BB)-2A-GFP | expressing Cas9 from *S. pyogenes* with 2A-EGFP, cloning backbone for sgRNA | Addgene (#48138), F. A. Ran et al. (12) |
| pSpCas9 (BB)-2A-GFP-sgRNA CAPN1 | expressing sgRNA for calpain 1 | This study |
| pSpCas9 (BB)-2A-GFP-sgRNA CAPN4 | expressing sgRNA for calpain 4 | This study |
| pSc-TIA-CMV-BSR-TIA | sgRNA to zebrafish *TIA* gene, CMV sequence and blasticidin resistance flanked by two TIA target sites | V. A. Blomen et al. (13) |
|  | | |
| **Oligonucleotide** | **Sequence (5´ to 3´)** | **Purpose** |
| SpeI-BGHpolyA-pLV | GAGGTTGATTATCATATGACTAGTCAACAGATGGCTGGCAACTAGAAGGCACAG | primer for amplification of constructs from pCMV-like Addgene vectors (e.g. pCMV-G-GECO) |
| PmeI-pCMV-pLV | GAGACTAGCCTCGAGGTTTAAACTCGAAATTAATACGACTCACTATAGG |  |
| SarAP1-R | GGTACCGATGCATCTTGCTCGATACATTTG | Amplification of SarAP1 promoter |
| SarAP1-F | GTCGACGCGGCCGCTGCATGCCTGATATTTTTG |  |
| attB1-*hla*-up-F | GGGGACAAGTTTGTACAAAAAAGCAGGCTTCCGCATCATTTGTTGTTAATAATG |  |
| *hla*-up-R | GATCGACCGCGGTAATGTAAATTATTTGTTCATGTACAAATAAATAT |  |
| *hla*-down-F | GATCGACCGCGGTTTCATCATCCTTCTATTTTTTAAAACGA |  |
| attB2-*hla*-down-R | GGGGACCACTTTGTACAAGAAAGCTGGGTTAATCGTTCTAAATCTAGCATCTTCTA |  |
| *hla*-test-f | ATATGTTTATTCATGATGTTGAC | primer to amplify the genomic locus of alpha-toxin gene (*hla*) |
| *hla*-test-r | AGAATTGGGAGTAGGAATG |  |
| Sense GIPZ | AATGATACGGCGACCACCGAGGACCGCGCACCTGGTGCATGAC |  |
| Reverse AGT | CAAGCAGAAGACGGCATACGAAGTCTAAAGTAGCCCCTTGAATTCCGAGGCAGTAG |  |
| Reverse TAC | CAAGCAGAAGACGGCATACGATACCTAAAGTAGCCCCTTGAATTCCGAGGCAGTAG |  |
| Reverse ATG | CAAGCAGAAGACGGCATACGAATGCTAAAGTAGCCCCTTGAATTCCGAGGCAGTAG |  |
| Reverse CGT | CAAGCAGAAGACGGCATACGACGTCTAAAGTAGCCCCTTGAATTCCGAGGCAGTAG |  |
| Reverse TGA | CAAGCAGAAGACGGCATACGATGACTAAAGTAGCCCCTTGAATTCCGAGGCAGTAG |  |
| Reverse GCA | CAAGCAGAAGACGGCATACGAGCACTAAAGTAGCCCCTTGAATTCCGAGGCAGTAG |  |
| Reverse GCA | CAAGCAGAAGACGGCATACGAGCACTAAAGTAGCCCCTTGAATTCCGAGGCAGTAG |  |
| Reverse GAC | CAAGCAGAAGACGGCATACGAGACCTAAAGTAGCCCCTTGAATTCCGAGGCAGTAG |  |
| RT-*hla*-fwd | GATCCTAACAAAGCAAGTTCTC | qRT-PCR for *hla* |
| RT-*hla*-rev | CAATTTGTTGAAGTCCAATG |  |
| RT-RNAIII-fwd | ACATAGCACTGAGTCCAAGG | qRT-PCR for RNAIII |
| RT-RNAIII-rev | TCGACACAGTGAACAAATTC |  |
| RT-*gyr*B-fwd | CGACTTTGATCTAGCGAAAG | qRT-PCR for *gyr*B |
| RT-*gyr*B-rev | ATAGCCTGCTTCAATTAACG |  |
| U6-fwd | GAGGGCCTATTTCCCATGATTCC | Sequencing of pSpCas9 (BB)-2A-GFP |
| CAPN1_sgRNA_top | caccGTCGGAGGAGATCATCACGC | sgRNA for knock-out of *CAPN1* |
| CAPN1_sgRNA_bottom | aaacGCGTGATGATCTCCTCCGAC |  |
| CAPN4_sgRNA_top | caccCATTGACACATGTCGCAGCA | sgRNA for knock-out of *CAPN4* |
| CAPN4_sgRNA_bottom | aaacATGATAAACTGGGGGTCAGC |  |

1. Grosz M, Kolter J, Paprotka K, Winkler AC, Schafer D, Chatterjee SS, Geiger T, Wolz C, Ohlsen K, Otto M, Rudel T, Sinha B, Fraunholz M. 2014. Cytoplasmic replication of *Staphylococcus aureus* upon phagosomal escape triggered by phenol-soluble modulin alpha. Cell Microbiol 16:451-65.

2. Giard DJ, Aaronson SA, Todaro GJ, Arnstein P, Kersey JH, Dosik H, Parks WP. 1973. In vitro cultivation of human tumors: establishment of cell lines derived from a series of solid tumors. J Natl Cancer Inst 51:1417-23.

3. Kreiswirth BN, Lofdahl S, Betley MJ, O'Reilly M, Schlievert PM, Bergdoll MS, Novick RP. 1983. The toxic shock syndrome exotoxin structural gene is not detectably transmitted by a prophage. Nature 305:709-12.

4. Carette JE, Raaben M, Wong AC, Herbert AS, Obernosterer G, Mulherkar N, Kuehne AI, Kranzusch PJ, Griffin AM, Ruthel G, Dal Cin P, Dye JM, Whelan SP, Chandran K, Brummelkamp TR. 2011. Ebola virus entry requires the cholesterol transporter Niemann-Pick C1. Nature 477:340-3.

5. Vann JM, Proctor RA. 1987. Ingestion of *Staphylococcus aureus* by bovine endothelial cells results in time- and inoculum-dependent damage to endothelial cell monolayers. Infect Immun 55:2155-63.

6. Fey PD, Endres JL, Yajjala VK, Widhelm TJ, Boissy RJ, Bose JL, Bayles KW. 2013. A genetic resource for rapid and comprehensive phenotype screening of nonessential *Staphylococcus aureus* genes. MBio 4:e00537-12.

7. Stelzner K, Hertlein T, Sroka A, Moldovan A, Paprotka A, Kessie DK, Mehling H, Potempa J, Ohlsen K, Fraunholz MJ, Rudel T. 2020. Intracellular *Staphylococcus aureus* employs the cysteine protease staphopain A to induce host cell death in epithelial cells. bioRxiv doi: 10.1101/2020.02.10.936575.

8. Liese J, Rooijakkers SH, van Strijp JA, Novick RP, Dustin ML. 2013. Intravital two-photon microscopy of host-pathogen interactions in a mouse model of *Staphylococcus aureus* skin abscess formation. Cell Microbiol 15:891-909.

9. Wiznerowicz M, Trono D. 2003. Conditional suppression of cellular genes: lentivirus vector-mediated drug-inducible RNA interference. J Virol 77:8957-61.

10. Zhao Y, Araki S, Wu J, Teramoto T, Chang YF, Nakano M, Abdelfattah AS, Fujiwara M, Ishihara T, Nagai T, Campbell RE. 2011. An expanded palette of genetically encoded Ca^2+^ indicators. Science 333:1888-91.

11. Wu J, Prole DL, Shen Y, Lin Z, Gnanasekaran A, Liu Y, Chen L, Zhou H, Chen SR, Usachev YM, Taylor CW, Campbell RE. 2014. Red fluorescent genetically encoded Ca2+ indicators for use in mitochondria and endoplasmic reticulum. Biochem J 464:13-22.

12. Ran FA, Hsu PD, Wright J, Agarwala V, Scott DA, Zhang F. 2013. Genome engineering using the CRISPR-Cas9 system. Nat Protoc 8:2281-2308.

13. Blomen VA, Majek P, Jae LT, Bigenzahn JW, Nieuwenhuis J, Staring J, Sacco R, van Diemen FR, Olk N, Stukalov A, Marceau C, Janssen H, Carette JE, Bennett KL, Colinge J, Superti-Furga G, Brummelkamp TR. 2015. Gene essentiality and synthetic lethality in haploid human cells. Science 350:1092-6.
